# Identification and functional characterization of three new terpene synthase genes involved in chemical defense and abiotic stresses in *Santalum album*

**DOI:** 10.1186/s12870-019-1720-3

**Published:** 2019-03-28

**Authors:** Xinhua Zhang, Meiyun Niu, Jaime A. Teixeira da Silva, Yueya Zhang, Yunfei Yuan, Yongxia Jia, Yangyang Xiao, Yuan Li, Lin Fang, Songjun Zeng, Guohua Ma

**Affiliations:** 1 Key Laboratory of South China Agricultural Plant Molecular Analysis and Genetic Improvement, South China Botanical Garden, Chinese Academy of Sciences, Guangzhou, China; 2 University of the Chinese Academy of Sciences, Beijing, China; 3 P. O. Box 7, cho post office, Ikenobe 3011-2, Kagawa-Ken, Miki, 761-0799 Japan

**Keywords:** Abiotic stress, Methyl jasmonate, Salicylic acid, *Santalum album*, Sesquiterpene, Terpene synthase

## Abstract

**Background:**

It is well known that aromatic essential oils extracted from the heartwood of *Santalum album* L. have wide economic value. However, little is known about the role of terpenoids in response to various adverse environmental stresses as other plants do in the form of signals during plant-environment interactions.

**Results:**

In this study, trace amounts of volatiles consisting of α-santalene, *epi*-β-santalene, β-santalene, α-santalol, β-santalol, (*E*)-α-bergamotene, (*E*)-β-farnesene and β-bisabolene were found in the leaves of mature *S. album* trees. We identified more than 40 candidate terpene synthase (TPS) unigenes by mining publicly-available RNA-seq data and characterized the enzymes encoded by three cDNAs: one mono-TPS catalyzes the formation of mostly α-terpineol, and two multifunctional sesqui-TPSs, one of which produces (*E*)-α-bergamotene and sesquisabinene as major products and another which catalyzes the formation of (*E*)-β-farnesene, (*E*)-nerolidol and (*E,E*)-farnesol as main products. Metabolite signatures and gene expression studies confirmed that santalol content is closely related with santalene synthase (SaSSY) transcripts in heartwood, which is key enzyme responsible for santalol biosynthesis. However, the expression of three new SaTPS genes differed significantly from *SaSSY* in the essential oil-producing heartwood. Increased activities of antioxidant enzymes, superoxide dismutase, catalase, peroxidase and ascorbate peroxidase, were detected in different tissues of *S. album* plants after applying 1 mM methyl jasmonate (MeJA) and 1 mM salicylic acid (SA), or exposure to 4°C, 38°C and high light intensity. MeJA and SA dramatically induced the expression of *SaTPS1* and *SaTPS2* in leaves. *SaTPS1* to *3* transcripts were differentially activated among different tissues under adverse temperature and light stresses. In contrast, almost all *SaSSY* transcripts decreased in response to these environmental stresses, unlike *SaTPS1* to *3*.

**Conclusions:**

Multifunctional enzymes were biochemically characterized, including one chloroplastic mono-TPS and two cytosolic sesqui-TPSs in sandalwood. Our results suggest the ecological importance of these three new SaTPS genes in defensive response to biotic attack and abiotic stresses in *S. album*.

**Electronic supplementary material:**

The online version of this article (10.1186/s12870-019-1720-3) contains supplementary material, which is available to authorized users.

## Background

Terpenoids constitute the largest and most diverse class of chemical compounds present in all living organisms. In particular, flowering plants exhibit an unusually high number of terpenoids for a variety of basic functions in growth and development, including hormones, components of electron transfer systems, reagents for protein modification, determinants of membrane fluidity, antioxidants, and others [1]. Different plant lineages also synthesize hundreds of distinct and specialized plant terpenoids. Traditionally, specialized terpenoids are used as natural flavor and aroma compounds and have a beneficial impact on humans as health-promoting compounds. Moreover, the ecological functions of terpenoids have gained increased attention [2].

All plant terpenes are made from the two simple five-carbon building blocks, isopentenyl diphosphate and its isomer, dimethylallyl diphosphate, both of which are derived from the mevalonate pathway in the cytosol or the methylerythritol phosphate pathway in plastids [3]. Condensation of the C5 precursors leads to the formation of prenyl diphosphates, geranyl diphosphate (GPP), farnesyl diphosphate (FPP) and geranylgeranyl diphosphate (GGPP), which are subsequently converted into monoterpenes, sesquiterpenes, and diterpenes by terpene synthases (TPSs), respectively. Terpenoid biosynthesis occurs within specific tissues or at specific developmental stages in plants, such as in resin ducts and glandular trichomes [4].

During recent decades, there has been major progress in the identification and functional characterization of genes and enzymes involved in terpenoid biosynthesis [5]. Many mono-TPS and sesqui-TPS genes have been reported from several plants, including Arabidopsis (*Arabidopsis thaliana*) [6], grapevine (*Vitis vinifera*) [7], tomato (*Solanum lycopersicum*) [8], poplar (*Populus trichocarpa*) [9], spruce (*Picea* spp.) [10], *Artemisia* spp. [11] and citrus (*Citrus sinensis*) [12]. An unusual feature of many TPSs is that they are able to produce terpenoids of structurally and stereochemically diverse compounds from a single substrate [13]. Based on sequence relatedness, functional assessment and gene architecture, the TPS gene family has been divided into eight subfamilies, designated as TPS-a through TPS-h [14, 15]. Class I contains TPS-c (copalyl diphospate synthases), TPS-e (*ent*-kaurene synthases), TPS-f (other di-TPSs), and TPS-h (lycopod-specific); Class II consists of TPS-d, which is gymnosperm-specific; Class III consists of angiosperm-specific TPS-a, TPS-b (cyclic mono-TPSs and hemi-TPSs), and TPS-g (acyclic mono-TPSs).

Plants produce a number of terpenoid metabolites for adaptation to adverse environments, including biotic and abiotic stresses [1]. Volatile terpenoids are inducibly emitted in response to herbivore or pathogen attacks in plants, and not only function directly as defensive phytoalexins for deterring detrimental attackers but also indirectly attract natural enemies of pathogens and herbivores [16]. Studies have shown that the plant hormones jasmonic acid (JA) and salicylic acid (SA) play important roles during biotic attack as defensive signaling [17, 18]. Therefore, exogenously applied JA and SA can be used as biotic stress mimics for studying the mechanism underlying the interaction between plants and biotic stresses [19, 20], and for increasing the production of plant terpenoids in specific organs to produce specialized terpenoids [21]. For example, in sweet wormwood (*Artemisia annua*), JA can induce the *AaMYC2* gene coding for a basic helix-loop-helix-type transcription factor that binds directly to G-box-like motifs within the promoters of two genes encoding multifunction cytochrome P450 monooxygenase (CYP71AV1) and artemisinic aldehyde delta-11 (13) reductase during artemisinin biosynthesis [22]. In *A. annua*, SA enhanced the expression level of amorpha-4,11-diene synthase and increased artemisinin production [23]. In agarwood (*Aquilaria sinensis*), exogenously applied methyl jasmonate (MeJA) in cell suspension cultures, callus and plant stems induced the biosynthesis and accumulation of sesquiterpene compounds, especially δ-guaiene [24–26].

Moreover, the emission of some biogenic terpenes can be induced by environmental factors such as temperature and light. High temperature was first recognized as one abiotic stress capable of inducing volatile terpenoids in slash pine (*Pinus elliottii*) [27]. In addition, the elevated emission of a variety of terpene volatiles, including monoterpenes and sesquiterpenes, from leaves, flowers and other organs in a range of woody and herbaceous species was observed under temperature, light, drought and salt stresses [28–34]. The production of terpenes was also induced by ozone, ultraviolet-B rays and γ-rays, which are oxidative stresses [35–37]. A common biochemical mechanism explaining the emission of volatile terpenoids induced by exogenous stresses claims that plants have the ability to synthesize terpenoids against oxidative damage resulting in elevated levels of reactive oxygen species (ROS) [1, 38].

More than 150 terpenoid compounds were identified from the essential oil of sandalwood (*Santalum album*) heartwood (HW) [39]. The major components were α- and β-santalol, but some minor compounds also exist, including α-santalene, β-santalene, α-bergamotene, and others. In recent years, key genes responsible for santalol biosynthesis have been functionally characterized, including santalene synthase (SaSSY) [40–42], SaCYP736A167 and SaCYP76AF39V1 [43, 44]. Another five TPSs, namely SamonoTPS1, SasesquiTPS1, Sasesquisabinene synthases (SaSQS1 and SaSQS2), and Sabisabolene synthase (SaBS), have also been identified [42, 45]. Kulheim et al. (2014) attempted to enhance the essential oil productivity of sandalwood by applying 0.01% MeJA to the leaves of seedling younger than one year [46]. Their findings indicate that the expression levels of only two genes coding for hydroxymethylglutaryl CoA reductase (HMCR) and farnesyl diphosphate synthase (FPPS) were slightly induced in leaves and stems of seedlings in response to MeJA treatment. Our previous study in *S. album* indicated that six *TPS* unigenes coding for (*E*)-β-ocimene/α-farnesene synthase, santalene/bergamotene synthase 1 (SS/BS), nerolidol synthase 1-like, myrcene synthase, geranyl linalool synthase, and SamonoTPS1 were differentially expressed in response to cold stress (4°C) [47]. However, little is known about the contribution of TPSs in response to various adverse environmental stresses in *S. album*.

In this study, transcriptomic data from leaves, stems and roots which were deposited at the Sequence Read Archive (SRC) [42, 44, 47, 48] were integrated to identify expressed members of the sandalwood TPS gene family. Of these, three *SaTPS* genes, designated as *SaTPS1* to *3*, were isolated and functionally characterized. The expression pattern and roles of *SaTPS1* to *3* besides SaSSY in response to MeJA, SA, and adverse temperature and light, were comparatively investigated. This study paves the way for revealing the regulatory mechanisms underlying the biosynthesis of terpenoids in biotic and abiotic stresses in *S. album*.

## Results

### Differences in content and composition of volatiles among four tissues

**Fig. 1:**
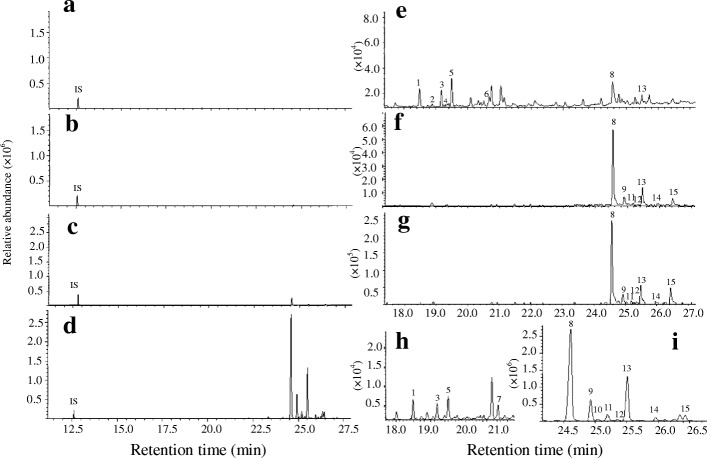
Sesquiterpenoid profile from different extracts. Sesquiterpenoids were identified against those in mass spectra reference libraries, NIST2005, NIST2005s, NIST2014, NIST2014s and FFNSC1.3 and a comparison of their retention indices. **a**, **e**: YL, young leaves, **b**, **f**: IW, immature wood, **c**, **g**: SW, sapwood, **d**, **h**, **i**: HW, heartwood. Segments (**e**, **f**, **g**, **h** and **i**) of the GC profile (**a**, **b**, **c**, **d**) are magnified in the right panel, respectively. Peaks: 1, α-santalene, 2, (*E*)-α-bergamotene, 3, *epi*-β-santalene, 4, (*E*)-β-farnesene, 5, β-santalene, 6, β-bisabolene, 7, unknown, 8, α-santalol, 9, α-*trans*-bergamotol, 10, α-santalol isomer, 11, *epi*-β-santalol, 12, unknown, 13, β-santalol, 14, β-santalol isomer, 15, lanceol. Peak numbers are indicated in order of elution from a HP-5MS column. IS, internal standard (*n*-dodecane)

To understand the distribution pattern and differences in components of *S. album* volatiles, we examined the metabolic compounds from pentane extracts of young leaves (YL), sapwood (SW) and HW from mature trees and immature wood (IW) from immature trees by GC-MS. There were significant differences in the volatile content among the four tissues. As shown in Fig. 1, the most abundant volatile was detected in HW (Fig. 1a-d). Major sesquiterpenols, α- and β-santalol, were detected in these four tissues, and sesquiterpenes, α- and β-santalene, and *epi*-β-santalene were identified in YL and HW, but not in IW and SW (Fig. 1e-i, Additional file 1: Table S1). It was curious that trace amounts of α- and β-santalene, *epi*-β-santalene and α-santalol (peaks 1, 3, 5 and 8, respectively) were found in almost the same proportion in YL (Fig. 1e). Of these, the distribution of the first three compounds matched that in HW (Fig. 1h, peaks 1, 3 and 5). Moreover, (*E*)-α-bergamotene, (*E*)-β-farnesene, and β-bisabolene were also detected from leaf extracts of *S. album*. Almost the same distribution of sesquiterpenols as those in SW and HW was observed in IH (Fig. 1 f, g and i, peaks 8–15), indicating that the accumulation of sandalwood oil occurs at an early developmental stage of the stems and that more essential oil is biosynthesized in HW as the sandalwood tree grows older.

### Identification of new terpene synthases in *S. album*

We combined transcriptome sets downloaded from NCBI, including transcripts from leaves, stems and roots as described in the Methods. D*e novo* assembly yielded 164,548 unigenes from the three tissues (Additional file 1: Figure S1, Additional file 1: Table S2). Many unigenes were identified as *SaTPS* genes, including 38 core terpene synthase genes and nine triterpene-specific synthase genes (Additional file 1: Table S3). Of these, six unigene sequences were completely aligned with those of *S. album* submitted to NCBI [40, 42, 45]. Three unigenes contained full-length ORFs encoding TPSs, designated as *SaTPS1* to *3*. In order to confirm that the assembled unigenes generated using the Trinity assembler were accurate, we designed primers to anneal around the predicted start and stop codons of unigenes to amplify ORFs of the three predicted *SaTPS* genes (Fig. S2). Subsequent sequencing of these nucleotides demonstrated that they were exactly as predicted by the unigene assembly.

### Sequence and phylogenetic analysis of three SaTPSs

**Fig. 2:**
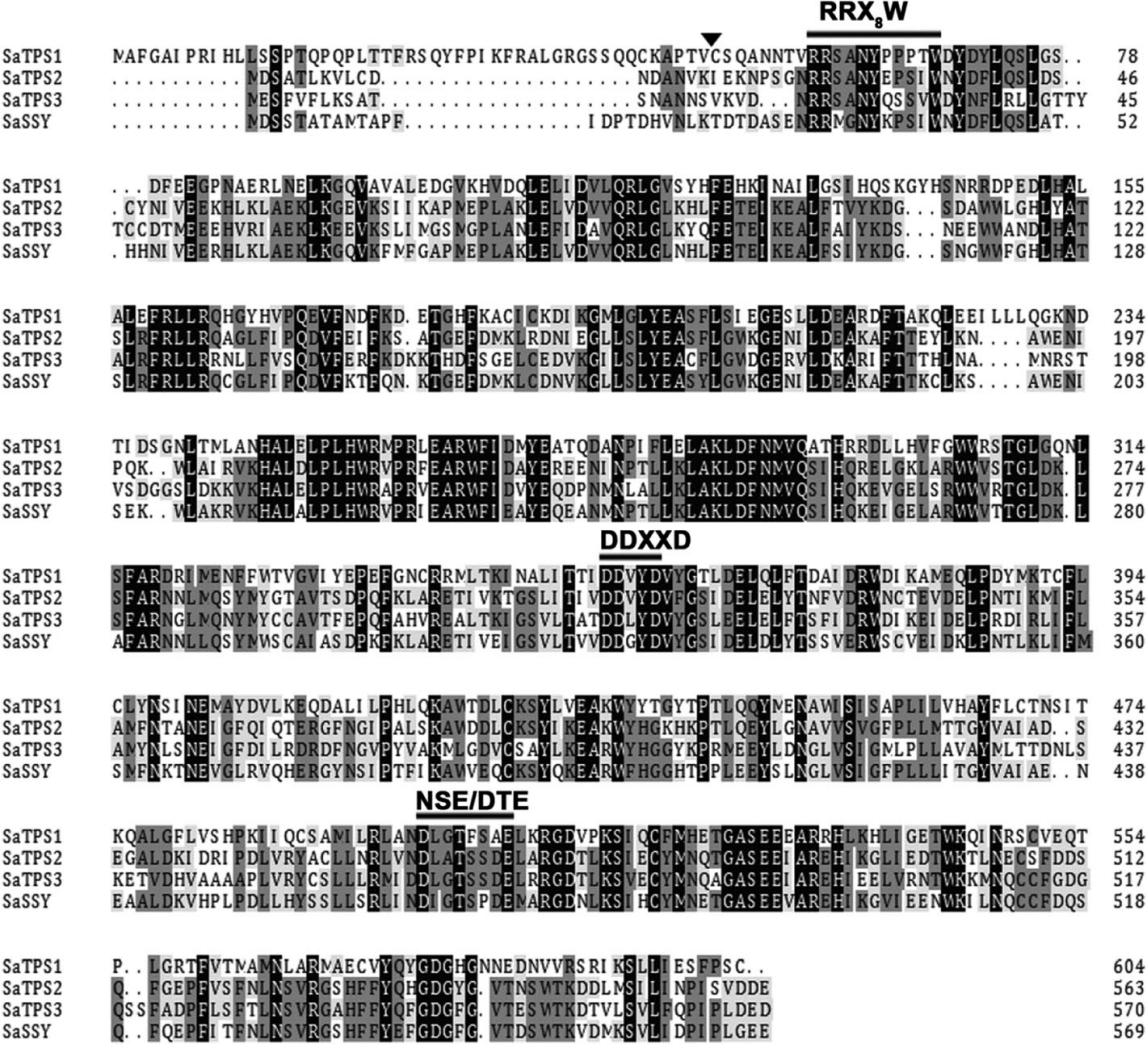
Comparison of deduced amino acid sequences of SaTPSs. The deduced amino acid sequences of *SaTPS* genes were aligned using DNAMAN 6.0 (Lynnon Biosoft, San Ramon, CA, USA). The Asp-rich domain DDXXD, the RRX8W motif, and the NSE/DTE motif, which are highly conserved in plant TPSs and required for TPS activity, are indicated. The arrowhead denotes the predicted cleavage site of plastidial transit peptide of SaTPS1. Completely conserved residues are shaded in black, identical residues in dark grey, and similar residues in light grey. Dashes indicate gaps introduced to maximize sequence alignment

Three predicted proteins encoded by *SaTPS1* to *3* with a range of 564 to 604 amino acids had predicted *pI*s of 6.01, 5.02 and 5.03, respectively (Additional file 1: Table S4). As shown in Fig. 2, SaTPS1 to 3 contain the arginine-tryptophan motif, R(R) X8W, which is conserved in most mono-TPSs and in some sesqui-TPSs near the N-terminus. The highly conserved aspartate-rich motif (DDXXD) and NSE/DTE motif, which are crucial for chelating divalent cations, typically Mg^2+^, in the C-terminal domain [49], were present in these three SaTPSs. One of the distinguishing structural features between mono- and sesqui-TPSs is the presence of an N-terminal plastid transit peptide sequence. Using the signal sequence analysis program ChloroP (http://www.cbs.dtu.dk/services/ChloroP), a putative N-terminal plastid transit peptide (Tp) sequence of 44 amino acids for SaTPS1 was predicted, indicating that it is likely a mono-TPS. However, SaTPS2 and SaTPS3 did not contain a plastid Tp sequence, suggesting that they are sesqui-TPSs (Additional file 1: Table S4).

**Fig. 3:**
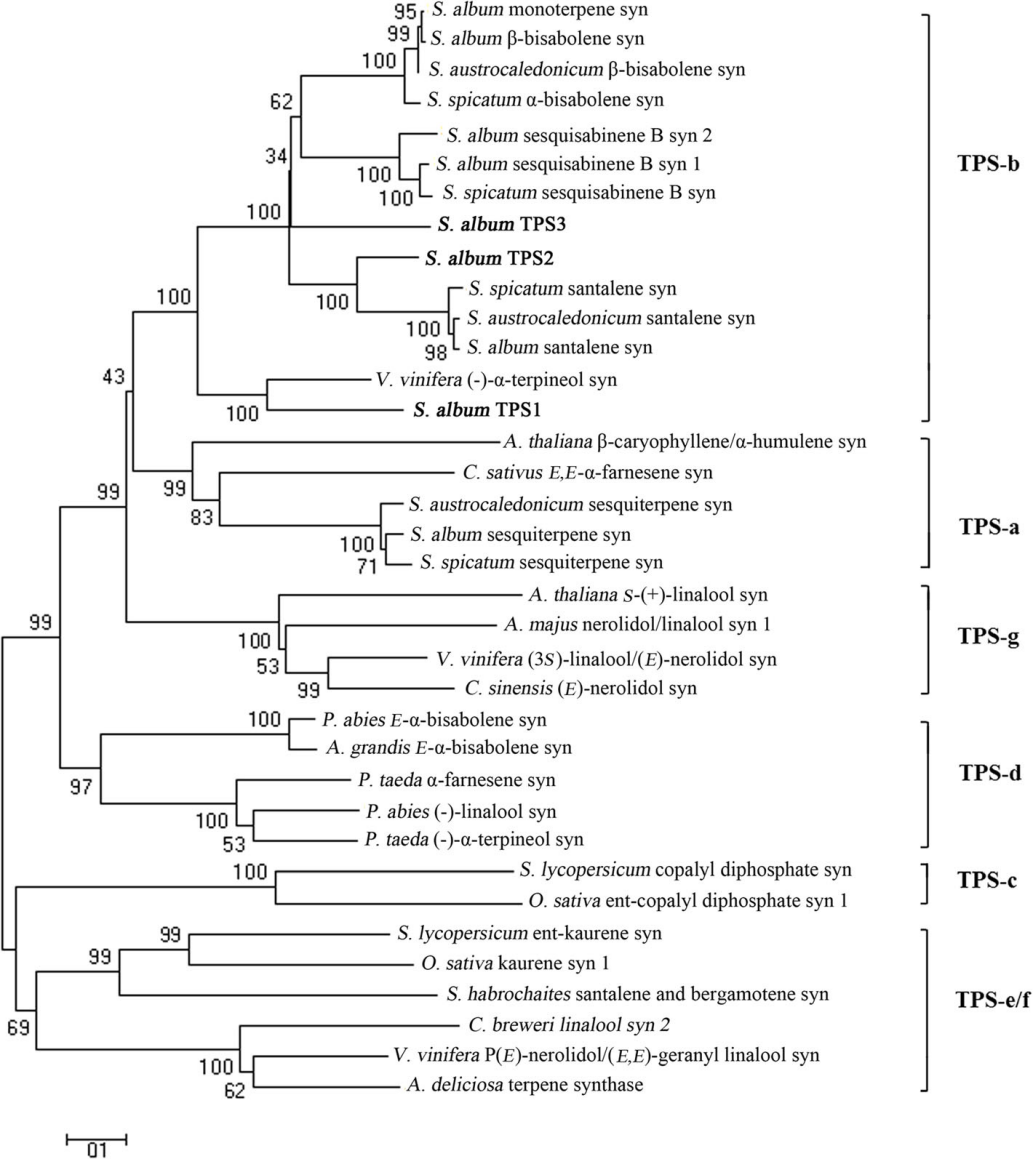
Phylogenetic positioning of *SaTPS* protein products within representative samples of known plant TPS. The neighbour-joining tree was drawn using the MEGA 6 program from an alignment of full-length SaTPSs with other plant TPSs [75]. The seven TPS subfamilies a-g on the right-hand side are delimited based on the taxonomic distribution of the TPS families [15]. Bootstrap values from 1000 replicates were used to assess the robustness of the trees. Names of organism TPSs (NCBI Protein no.) can be retrieved from Additional file 1: Table S4

A phylogenetic tree based on the deduced amino acid of sandalwood TPSs and other plant species showed that all *Santalum* TPSs clustered in the TPS-b clade, with the exception of SasesquiTPS and sesquiTPSs in *S. spicatum* (SspicsesquiTPS) and *S. austrocaledonicum* (SaustsesquiTPS) (Fig. 3; Additional file 1: Table S5), among which the majority of angiospermous monoterpene synthases reside [15]. The most similar TPS sequence to SaTPS1 is that of (−)-α-terpineol synthase from *V. vinifera* with 63% identity (Additional file 1: Figure S3; Additional file 1: Table S6) (Martin and Bohlmann, 2004). SaTPS2 clustered most closely to SaSSY, SspicSSY and SaustSSY, with a 100% bootstrap (Jones et al., 2011), and its amino acid sequence had highest identity with SaspiSSY (72%). SaTPS3 formed a clade with several previously characterized TPSs from *Santalum* species, including SaMonoTPS, SaSQS1, SaSQS2, SaBS, SspicBS and Sspicsesquisabinene [42, 45, 50]. Bioinformatics and phylogenetic analyses suggests that SaTPS2 and SaTPS3 might be involved in the production of essential oil in *S. album*.

### Expression patterns of three *SaTPS* genes differ from *SaSSY*

**Fig. 4:**
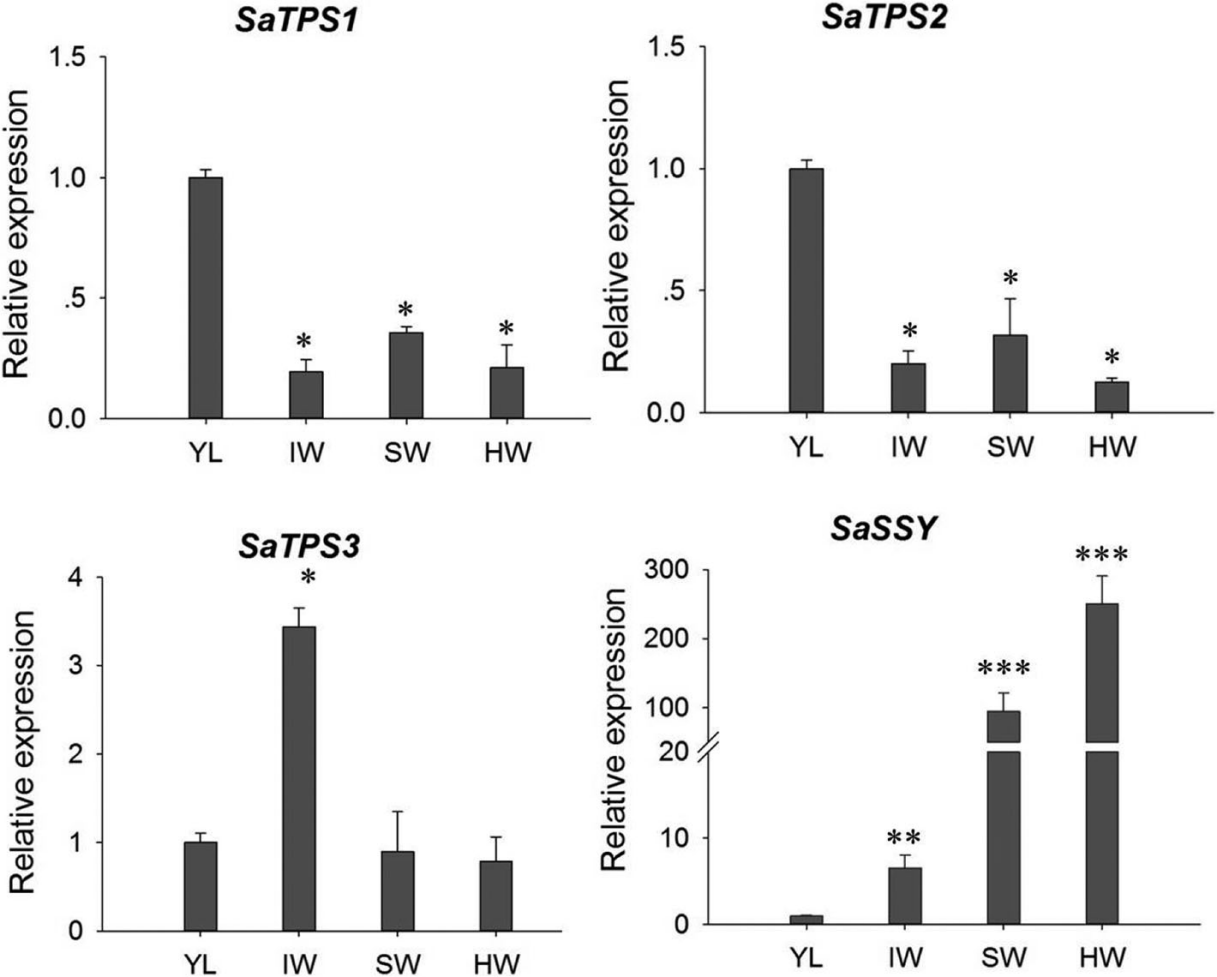
Expression levels of *SaTPS1* to *3* genes in different tissues. YL, young leaves; IW, immature wood; SW, sapwood; HW, heartwood. Significant differences were assessed by a student’s *t*-test: *P < 0.05, **P < 0.01 and ***P < 0.001

The tissue-specific expression of *SaTPS1* to *3* genes was determined by qRT-PCR in four key tissues, i.e., YL, IW, SW and HW. The transcript abundance of *SaTPS1* and *SaTPS2* genes showed a similar expression pattern, with highest expression in YL (Fig. 4). *SaTPS3* showed three-fold higher expression in IW than in YL, SW and HW. In contrast, *SaSSY* was 100- and 250-fold higher in SW and HW, respectively. Additionally, comparisons of the expression levels among these *SaTPSs* in specific tissues showed that *SaTPS3* showed higher expression than *SaTPS1* and Sa*TPS2* in the four corresponding tissues (Additional file 1 :Figure S4). Of note, the level of *SaSSY* transcript showed a 200- and 1200-fold increase relative to *SaTPS1* and *SaTPS2* in SW and HW, respectively. These results suggest that *SaTPS3* might play a role in the accumulation of HW essential oil whereas *SaTPS1* and *SaTPS2* play a minor role in the formation of terpenoids in *S. album* HW.

### Subcellular localization of SaTPSs

**Fig. 5:**
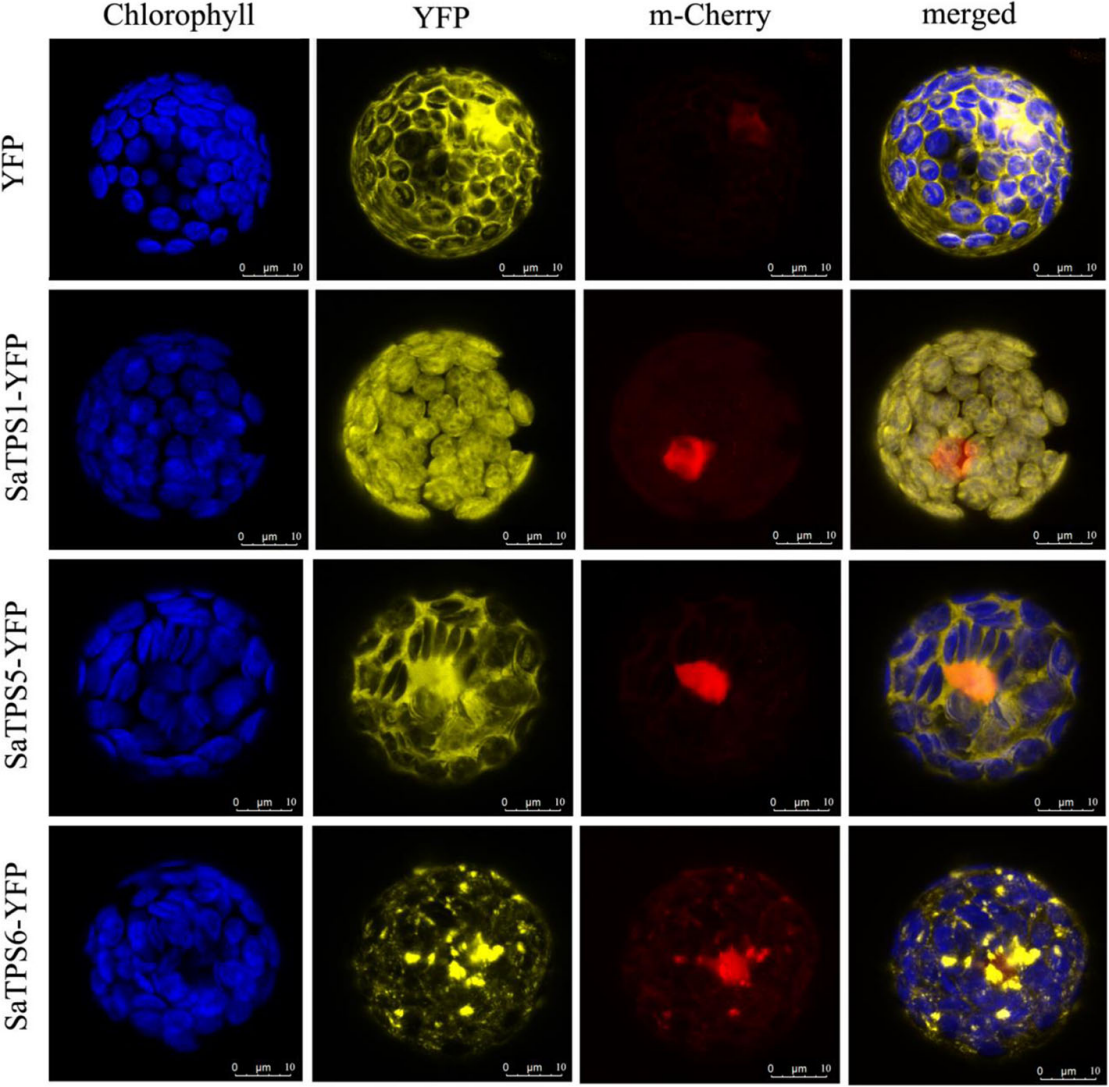
Subcellular localization of SaTPS1 to 3 in Arabidopsis mesophyll protoplasts. Protoplasts were transiently transformed with SaTPS-YFP constructs or YFP vector using a modified polyethylene glycol method. YFP fluorescence was observed with a laser scanning confocal microscope. Yellow fluorescence indicates SaTPS-YFP fusion protein signal. Blue signal indicates chlorophyll (Chl) autofluorescence and red signal indicates m-Cherry fluorescence. The merged images represent a digital combination of Chl autofluorescence, YFP fluorescent and m-Cherry protein fluorescence images. Fluorescence was excited for YFP at 514 nm, for Chl at 543 nm and for m-Cherry at 587 nm. Scale bar = 10 μm

To elucidate the function of SaTPS1 to 3, subcellular localization of each SaTPS-YFP (yellow fluorescent protein) fusion protein provided preliminary evidence. Phylogenetic and bioinformatics-based analyses in an attempt to classify TPSs indicated that SaTPS1-YFP, which has the N-terminal plastid transit peptide sequence, was localized in chloroplasts, whereas SaTPS2-YFP and SaTPS3-YFP were distributed throughout the cytosol (Fig. 5). Based on the results of subcellular localization, it was concluded that SaTPS1 is likely involved in monoterpene biosynthesis in plastids, whereas SaTPS2 and SaTPS3 might produce sesquiterpenes in the cytosol.

### Functional characterization of the enzymes encoded by *SaTPS1* to *3*

**Fig. 6:**
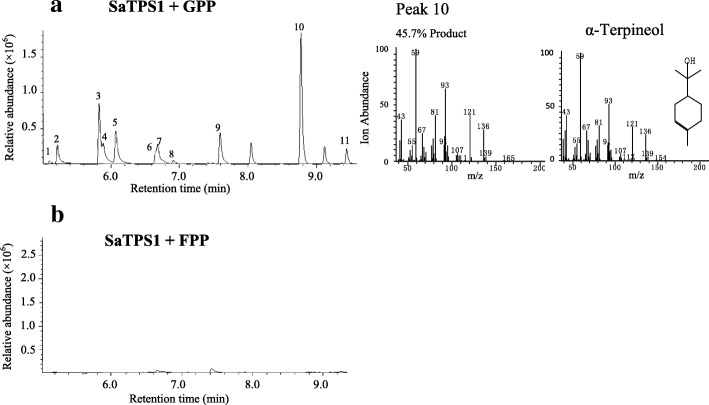
In vitro enzymatic assays of recombinant SaTPS1. In vitro enzyme assays of recombinant SaTPS1 using GPP (**a**) and FPP (**b**) as the substrate in the presence of Mg^2+^. The reaction products were analyzed by GC-MS. The peaks marked with numbers were identified by comparison of their mass spectra with those in the library data and comparison of their retention index. Peaks: 1, α-thujene, 2, α-pinene, 3, sabinene, 4, β-pinene, 5, myrcene, 6, limonene, 7, cineole, 8, β-ocimene, 9, linalool, 10, α-terpineol, 11, geraniol. Mass spectra for the major product and corresponding authentic standard are shown on the right side of the figure. *m/z*, mass-to-charge ratio

All cDNAs subcloned into pET28a vector with 6His-tagged were successfully expressed in *E. coli* Rosetta 2 (DE3) competent cells and recombinant proteins were then purified with a Ni-NTA agarose affinity column (Additional file 1 :Figure S5). We found that this cDNA without the transit peptide sequence could improve the expression of *SaTPS1* in *E. coli*. Following an in vitro enzyme activity assay, *SaTPS1* was confirmed to encode a mono-TPS enzyme that catalyzed the formation of mostly α-terpineol (45.7%), while sabinene (14.9%), linalool (11.7%) and myrcene (10.8%), as well as a few minor monoterpenes, were also produced with GPP as the substrate and Mg^2+^, as detected by GC-MS analysis (Fig. 6a, Additional file 1: Table S7). Although the same compounds were produced when Mg^2+^ was replaced by Mn^2+^, levels of product formation were 5-fold lower and the three major compounds, linalool (35.8%), α-terpineol (25.7%), and geraniol (12.9%) were formed (Additional file 1: Figure S6, Additional file 1: Table S7). No terpene product was observed in the reaction of SaTPS1 with FPP as a substrate and Mg^2+^ or Mn^2+^ (Fig. 6b). This enzyme was designated as α-terpineol synthase because it is a major monoterpene product and due to its preference for the metal ion, Mg^2+^.

**Fig. 7:**
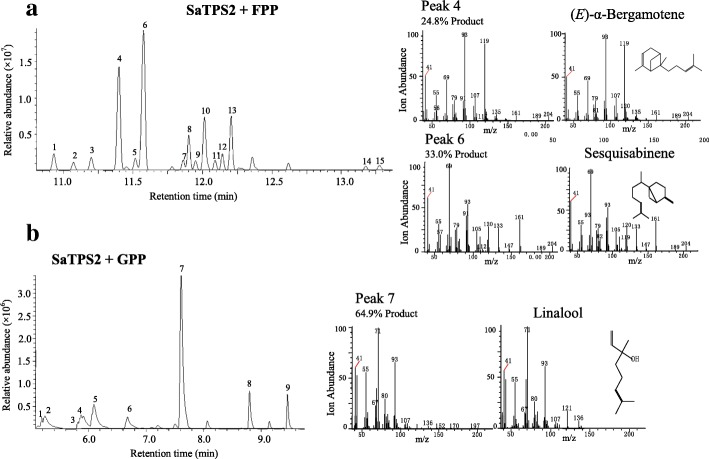
In vitro enzymatic assays of recombinant SaTPS2. In vitro enzyme assays of recombinant SaTPS2 using FPP (**a**) or GPP (**b**) as the substrate in the presence of Mg^2+^. The reaction products were analysed by GC-MS. Peaks marked with numbers were identified by comparison of their mass spectra with those in the library data and a comparison of their retention index. Peaks in **a**: 1, 7-*epi*-sesquithujene, 2, unknown, 3, α-bergamotene isomer, 4, (*E*)-α-bergamotene, 5, (*E*)-β-farnesene, 6, sesquisabinene, 7, unknown, 8, α-zingiberene, 9, α-bisabolene, 10, β-bisabolene, 11, γ-bisabolene, 12, unknown, 13, γ-bisabolene isomer, 14, β-bisabolol, 15, α-bisabolol. Peaks in **b**: 1, α-thujene, 2, α-pinene, 3, sabinene, 4, β-pinene, 5, myrcene, 6, limonene, 7, linalool, 8, α-terpineol, 9, geraniol. Mass spectra for the major products and corresponding authentic standards are shown on the right side of the figure, respectively. *m/z*, mass-to-charge ratio

GC-MS analysis of the reaction products catalyzed by SaTPS2 with FPP as a substrate in the presence of Mg^2+^ identified at least 15 sesquiterpenoids, with (*E*)-α-bergamotene (24.8%) and sesquisabinene (33.0%) as the two major products, β-bisabolene (9.0%), γ-bisabolene isomer (7.9%), and minor compounds, 7-*epi*-sesquithujene, (*E*)-β-farnesene, α-zingiberene, and others at relative proportions less than 5% (Fig. 7a; Additional file 1: Table S7). A similar product profile comprising the major compounds, (*E*)-α-bergamotene (22.4%) and sesquisabinene (35.6%), were detected when Mg^2+^ was replaced with Mn^2+^, but overall yield was relatively lower (Additional file 1: Figure S7, Additional file 1: Table S7). Incubation of SaTPS2 with GPP as a substrate and Mg^2+^ ion led to the production of nine monoterpenes (Fig. 7b; Additional file 1: Table S7). The most abundant compound was linalool (64.9%). Likewise, assays with Mn^2+^ instead of Mg^2+^ showed that almost the same compounds were observed, but overall yield was 5-fold lower (Additional file 1: Figure S8, Additional file 1: Table S7). Taken together, these results indicate that SaTPS2 is a multifunctional sesqui-TPS producing major (*E*)-α-bergamotene and sesquisabinene in the cytosol.

**Fig. 8:**
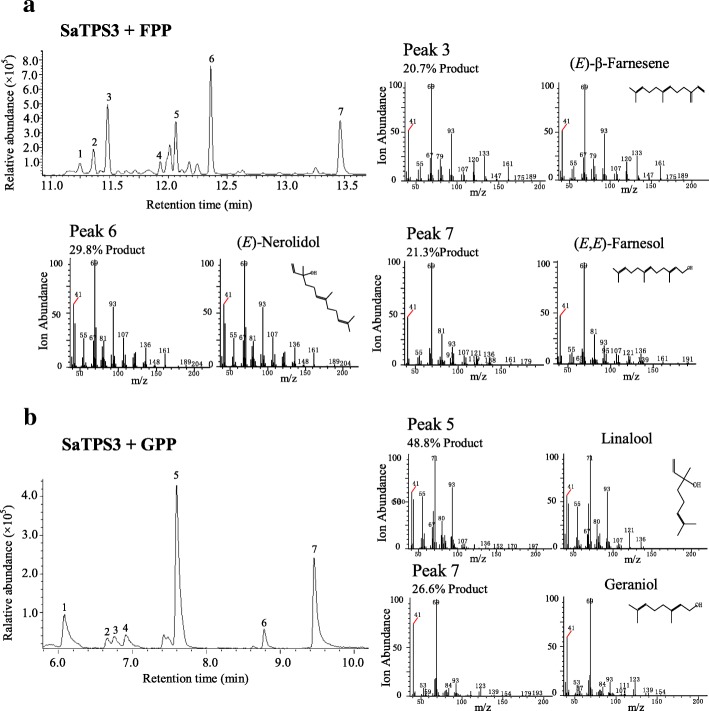
In vitro enzymatic assays of recombinant SaTPS3. In vitro enzyme assays of recombinant SaTPS3 using FPP (**a**) or GPP (**b**) as the substrate in the presence of Mg^2+^. The reaction products were analysed by GC-MS. The peaks marked with numbers were identified by comparison of their mass spectra with those in library data and a comparison of their retention index. Peaks in **a**: 1, cedrene, 2, unknown, 3, (*E*)-β-farnesene, 4, unknown, 5, γ-bisabolene, 6, (*E*)-nerolidol, 7, (*E,E*)-farnesol. Peaks in **b**: 1, myrcene, 2, limonene, 3, unknown, 4, β-ocimene, 5, linalool, 6, α-terpineol, 7, geraniol. Mass spectra for the major products and corresponding authentic standards are shown, respectively. *m/z*, mass-to-charge ratio

SaTPS3 converts FPP and GPP substrates to sesqui- and monoterpene products, respectively. When assayed with FPP and Mg^2+^, recombinant SaTPS3 catalyzed the synthesis of seven compounds, including (*E*)-β-farnesene (20.7%), (*E*)-nerolidol (29.8%) and (*E,E*)-farnesol (21.3%) as the three major products, while a high proportion of γ-bisabolene (13.8%) was also produced (Fig. 8a, Additional file 1: Table S7). In the presence of Mn^2+^, SaTPS3 recombination enzyme showed no activity, suggesting that SaTPS3 prefers Mg^2+^ in the reaction with FPP. Analysis of the reaction products formed after incubation of SaTPS3 with GPP showed that the main product linalool (44.8–53.3%) was formed with Mg^2+^ or Mn^2+^ in considerable yield (Fig. 8b, Additional file 1: Figure S9, Additional file 1: Table S7). For the three SaTPSs tested in this study, extracts prepared from *E. coli* transformed with PET28a lacking a cDNA insert and heat-denatured enzyme preparations served as controls, and no terpene products were observed. In all cases, expressed proteins incubated with GGPP did not produce detectable products.

The *K*_*m*_ value of purified SaTPS1 with GPP was 9.08 ± 1.26 μM and the *K*_*cat*_/*K*_*m*_ value was 0.0171 (Table 1; Additional file 1 :Figure S10). The SaTPS2 and SaTPS3 enzymes not only accept substrate FPP but also accept the precursor of monoterpenes, GPP, as analyzed above. Enzyme kinetic properties of SaTPS2 showed that catalysis with FPP and GPP resulted in a similar *K*_*m*_ and *K*_*cat*_/*K*_*m*_. The *K*_*m*_ value of the recombination SaTPS3 with FPP was 16.29 ± 1.48 μM, which was slightly higher than with GPP. The *K*_cat_/*K*_m_ with FPP was relatively lower than with GPP, indicating that the efficiency of this sesqui-TPS catalysis is higher when incubated with GPP.

**Table 1:** Enzyme kinetic properties of three SaTPSs

Enzyme	Substrate	*K*_*m*_ (μM)	*K*_*cat*_ (s^−1^)	*k*_cat_/*K*_m_ (s^−1^ μM^− 1^)
SaTPS1	GPP	9.08 ± 1.26	0.155 ± 0.007	0.0171
SaTPS2	FPP	12.15 ± 1.32	0.200 ± 0.008	0.0164
	GPP	12.27 ± 1.32	0.189 ± 0.007	0.0154
SaTPS3	FPP	16.29 ± 1.48	0.171 ± 0.006	0.0105
	GPP	14.38 ± 1.20	0.257 ± 0.008	0.0179

Values for FPP and GPP were measured in the presence of 10 mM Mg^2+^

All values represent mean ± SE, *n* = 3. *K*_*m*_, Michaelis constant; *k*_*cat*_, turnover

### Elevated antioxidant enzyme activity under multiple stress treatments

**Fig. 9:**
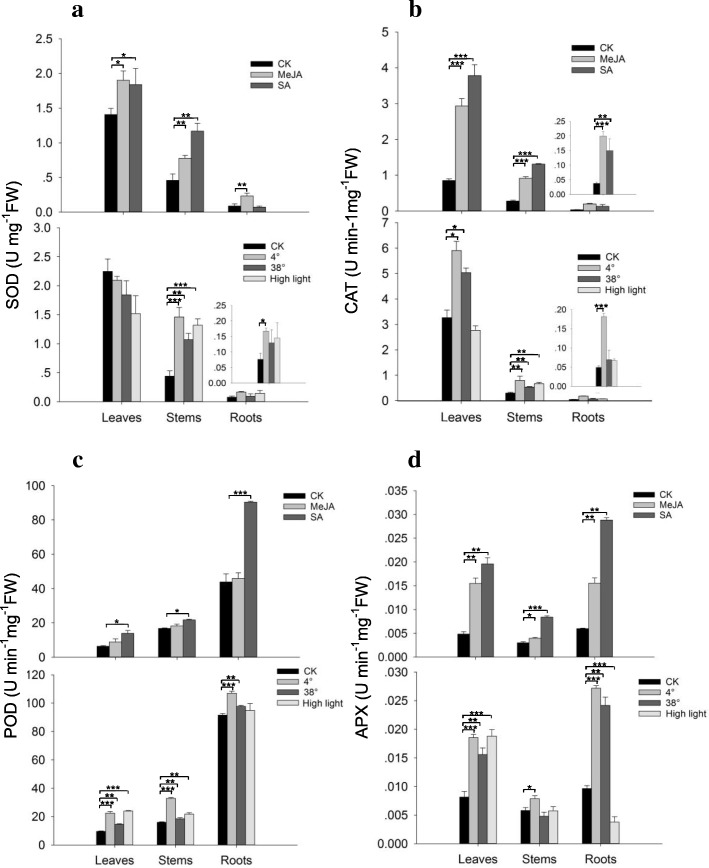
Effects of external stresses on activities of antioxidant enzymes in *S. album*. Six-month-old seedlings were sprayed with 1 mM MeJA or SA for 24 h or exposed to 4°C, 38°C or high light intensity (approx. 250 μmol m^− 2^ s^− 1^) for 12 h. Three measurements were averaged from the results of three replicated experiments. **a**: SOD, superoxide dismutase; **b**: CAT, catalase; **c**: POD, peroxidase; **d**: APX, ascorbate peroxidase. L, leaves; S, stems; R, roots. Significant differences were analyzed by a student’s *t*-test and indicated as *P < 0.05, **P < 0.01 and ***P < 0.001

The phytohormones JA and SA play crucial roles in regulating the defensive signaling network by elevating the levels of ROS [51, 52]. Moreover, the mechanism by which terpenes alleviate abiotic stress suggests a general antioxidant mechanism by which harmful ROS can be quenched by reacting with unsaturated isoprene, monoterpenes, and sesquiterpenes [38]. In our study, the activity of superoxide dismutase (SOD), a key contributor in the conversion of O^−^ to H_2_O_2_ in the presence of elevated levels of ROS, increased significantly under all treatments, except for a decrease in leaves exposed to 4°C, 38°C and high light intensity (approx. 250 μmol m^− 2^ s^− 1^) (Fig. 9a). Catalase (CAT) plays an important role in the antioxidant system because it enables plants to eliminate H_2_O_2_ by converting H_2_O_2_ into O_2_ and H_2_O (Miller et al., 2008). CAT activity was strongly elevated by these two elicitors. When the three tissues were exposed to temperature and light stresses, CAT activity was upregulated, the only exception being a slight decreased in leaves in response to high light intensity (Fig. 9b).

Similarly, peroxidase (POD), which detoxifies H_2_O_2_ in the chloroplasts and cytosol of plant cells, also constitutes the main H_2_O_2_-scavenging system in cells [53]. POD activity increased in all treatments and roots had two-fold higher POD activity than leaves (Fig. 9c). Conversely, SOD and CAT activities were relatively higher in leaves than in stems and roots, independent of whether they were treated or untreated, suggesting differential endogenous activities of SOD, CAT or POD among different tissues. Ascorbate peroxidase (APX) plays a key role in protecting plants against oxidative stress by scavenging H_2_O_2_ in different cell compartments [54]. As shown in Fig. 9d, APX activity increased significantly in most tissues under MeJA, SA, cold, heat and high light intensity treatments. Thus, the increase in SOD, CAT, POD and APX activities indicate that *S. album* seedlings respond to external stresses to protect cellular membranes against oxidative stress, suggesting that these enzymes play roles in tolerance to elicitors and environmental stresses in *S. album*. In addition, the effect of different adverse stresses on antioxidant enzyme activities will vary among tissues.

### Activation of *SaTPS1* to *3* gene expression in response to MeJA and SA

**Fig. 10:**
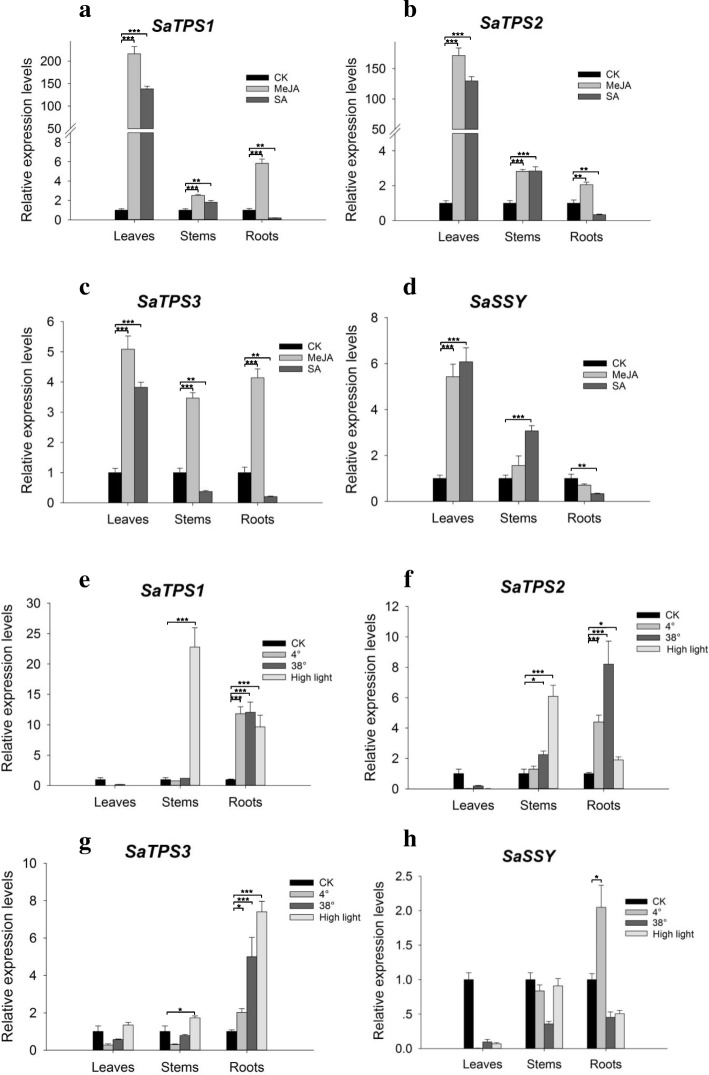
Transcript accumulation of *SaTPS1* to *3* under multiple stresses in *S. album*. (**a**-**d**) Six-month-old seedlings were sprayed with 1 mM MeJA or SA for 24 h. (**e**-**h**) Six-month-old seedlings were exposed to 4°C, 38°C or high light intensity (approx. 250 μmol m^− 2^ s^− 1^) for 12 h. Three measurements were averaged from the results of three replicated experiments. L, leaves; S, stems; R, roots. Significant differences were analyzed by a student’s *t*-test and indicated as *P < 0.05, **P < 0.01 and ***P < 0.001

To explore the responses of the three *SaTPS* genes under elicitors as well as temperature and high light intensity treatments, transcript levels for *SaTPS1* to *3* and *SaSSY* were determined by qRT-PCR. *SaTPS1* and *SaTPS2* exhibited similar expression patterns in response to the two elicitors. As shown in Fig. 10a and b, exogenous MeJA and SA dramatically induced the expression of *SaTPS1* and *SaTPS2* genes in leaves at 24 h, with more than a 170- and 130-fold increase, respectively, when compared with the controls. *SaTPS1* and *SaTPS2* transcripts also increased significantly in stems and roots after the application of MeJA. *SaTPS3* expression was activated in leaves, stems and roots with an approximately 5-, 3-, and 4-fold increase, respectively compared to the corresponding controls (Fig. 10c). In contrast, *SaTPS3* was significantly up-regulated in leaves by SA, but decreased in stems and roots. Moreover, the accumulation of *SaSSY* transcript were similar to that of *SaTPS3* in leaves under MeJA and SA treatments. However, the two exogenous hormones led to a decrease in *SaSSY* expression in roots compared with the control (Fig. 10d).

### Adverse temperature and high light induced differential accumulation of *SaTPS1*, *SaTPS2* and *SaTPS3*

*SaTPS1* to *3* expression levels were inhibited in leaves at 4°C and 38°C, but were significantly activated in roots when compared to the controls (Fig. 10e-g), suggesting that oxidative damage in leaves was more severe than in stems and roots. When exposed to high light intensity, *SaTPS1* and *SaTPS2* transcripts showed higher expression in stems and roots than in leaves, particularly *SaTPS1*, with a 25-fold increase in stems. The level of *SaTPS3* transcripts was highest in roots under 4°C, 38°C and high light intensity stresses, with approximately 2-, 6-, and 8-fold increases, respectively, compared to the control. Conversely, *SaSSY* transcript only increased by about 2-fold in roots at 4°C compared to the control. In all other cases, the expression level of *SaSSY* was down-regulated to some extent in all tissues examined (Fig. 10h).

## Discussion

### Candidate *TPS* genes from *S. album* by RNA-seq

Next generation sequencing has emerged as a promising platform to discover novel genes, enzymes, and molecular markers from non-model plant species. In recent years, transcriptomic approaches have been widely used to mine genes of terpenoid metabolism in *Santalum*. Research has mainly focused on cloning and characterization of SaTPSs, including SamonoTPS, SasesquiTPS, SaSSy, SauSSY, SspiSSY, SaSQS1, SaSQS2, SaBS, SauBS, SspiBS, and SspiTPS4 [40, 42, 45, 50]. Celedon et al. (2016) [44] found more than 30 *SaTPS* transcripts from a HW-specific transcriptome. In the present study, a set of *SaTPSs* was identified based on a combination of transcriptome data from leaves, stems and roots, implying that *S. album* likely has a mid-sized *TPS* gene family according to the classification of other angiosperm TPSs [15]. Currently, the genome assembly data of *S. album* is available [55], and we are eager to obtain the annotated genome so as to assess the exact size of TPSs in the *S. album* TPS family. Additionally, some TPSs specific to different tissues were detected (Additional file 1: Table S3), inferring that SaTPSs might play different roles in different tissues.

### Santalol content is closely related with *SaSSY* transcripts in heartwood

The major components of the total essential oil in the heartwood of *S. album* are α- and β-santalol, contributing over 80% of relative content [39]. SaSSY is a key enzyme responsible for the biosynthesis of α- and β-santalol [40–42]. Our study’s findings are in agreement with previous studies that showed that sandalwood essential oil accumulates in stem HW, followed by SW [44, 45]. However, α- and β-santalene and *epi*-β-santalene were not detected in SW, most likely due to undetectable or low amounts. To our knowledge, trace levels of volatiles consisting of α- and β-santalene, *epi*-β-santalene, α- and β-santalol, and other compounds in the leaves of *S. album* were first reported in this study. The distribution pattern of sesquiterpenols similar to those in SW and HW was found in IH (Fig. 1f, g and i). Collectively, these results established a foundation to reveal the mechanism of spatial and temporal distribution of volatiles in *S. album*. Moreover, high expression levels of *SaSSY* in the HW of *S. album* confirm that santalol content is closely related with *SaSSY* transcript level (Figs. 1 and 4).

In contrast, *SaTPS1* to *3* genes had relatively high expression levels in leaves (Fig. 4 and S4a) and trace amounts of (*E*)-α-bergamotene, (*E*)-β-farnesene and β-bisabolene in the leaves of mature *S. album* trees were also detected, suggesting that SaTPS2 and SaTPS3 might convert FPP to these sesquiterpenes, which belonged to major products when incubated with FPP in in vitro enzyme assays (Fig. 7a, 8a and Additional file 1: Table S7). In addition, major α-terpineol catalyzed by SaTPS1 was not identified in leaf extracts, inferring that it might be easily volatilized or that external stimuli might be required for its emission, aspects that still need to be explored.

### *SaTPS1* is likely a paralogous gene of *SamonoTPS1*

A study by Jones et al. (2008) [45] documented that SamonoTPS1 is a monoterpene synthase which produced a mixture of eight compounds, with α-terpineol (38.7%) and limonene (35.3%) as the two main products when incubated with GPP. In angiosperms, α-terpineol synthase from *V. vinifera* was reported as a monoterpene synthase, producing 14 compounds, including major monoterpene, α-terpineol (50.1%), 1,8-cineol (11.8%), β-pinene (8.5%), etc. [56]. Gao et al. (2018) [57] reported that FhTPS2 in flowers of *Freesia* × *hybrida* mainly converted GPP into α-terpineol (78.7%) and a few other monoterpenes, such as 1,8-cineole (6.9%), D-limonene (3.9%), α-pinene (3.2%), etc. In our study, SaTPS1 showed higher similarity at the nucleotide level with α-terpineol synthase sequences in *V. vinifera* than SamonoTPS1, and both of them have an N-terminal plastid transit peptide sequence and are closely related (Fig. 3; Additional file 1: Figure S3, Additional file 1: Table S6). Our biochemical characterization and subcellular localization studies confirm that SaTPS1 is a mono-TPS, α-terpineol synthase. Collectively, these results imply that *SaTPS1* may be a paralogous gene of *SamonoTPS1*.

### Functional divergence of TPSs has occurred in *Santalum*

SaSQS1 and SaSQS2 catalyze the cyclization of (*E,E*)-FPP to a single product, sesquisabinene [42]. SspiTPS4 catalyzed the formation of the most abundant sesquisabinene B (58%) in *S. spicatum* [50]. In this study, although a phylogenetic tree showed that SaTPS2 is much closer to SaSSY, SspicSSY and SaustSSY than to SaSQS1, SaSQS2 and SspiTPS4, in vitro enzyme assays of recombinant SaTPS2 and subcellular localization revealed that the mRNA of *SaTPS2* encodes a functional sesqui-TPS producing (*E*)-α-bergamotene (24.8%) and sesquisabinene (33.0%) as two major products. Moreover, both recombinant SaBS and SauBS can react with FPP to produce β-bisabolene as a major product [40, 42]. SspiBS produced a mixture of β-bisabolene and α-bisabolol, along with traces of α-bisabolene and farnesene isomers [40]. SaTPS2 could synthesize a relatively large amount of β-bisabolene (9.0%) and γ-bisabolene (1.6%) (Additional file 1: Table S7). Taken together, these results suggest that functional divergence of TPS has occurred in *Santalum* species. The diversification of terpenoid biosynthesis has also been reported in *Oryza TPS* genes [58].

In angiosperms, only the maize gene *TPS1* produces (*E*)-β-farnesene (26%), (*E*)-nerolidol (29%), and (*E*,*E*)-farnesol (45%) as the only three products in vitro [59]. Phylogenetically, SaTPS3 is paraphyletic group with other TPSs of *Santalum* spp. in the TPS-b group (Fig. 3). The function of SaTPS3 is similar to TPS1 in maize where it catalyzes the formation of three major compounds, i.e. (*E*)-β-farnesene (20.7%), (*E*)-nerolidol (29.8%) and (*E,E*)-farnesol (21.3%). However, SaTPS3 additionally synthesized γ-bisabolene, cedrene and two unknown sesquiterpene compounds in relative amounts that exceeded 28%, suggesting that SaTPS3 is not a highly conserved TPS in angiosperms. Diversity of function may be an evolution of adaptive selection in monocotyledons and dicotyledons.

### *SaTPS1* to *3* play important roles in chemical defense

The inductive roles played by JA- and SA-induced biosynthesis of terpenoids have also been widely demonstrated in recent years, playing similar roles as those during attack induced by fungal elicitation and mechanical wounding [60–62]. For example, the transcript level of *TPS1* producing (*E*)-β-farnesene, (*E*)-nerolidol and (*E*,*E*)-farnesol in vitro in maize cv. B73 was elevated after herbivory, or after mechanical damage with and without treatment with 5 μl of Egyptian cotton leafworm regurgitant or 5 μl of volicitin [59]. The volatile (*E*)-α-bergamotene is a well-characterized defensive compound in wounded leaves induced by herbivores [63] and in flowers [64, 65] of *Nicotiana attenuata* induced by JA-mediated signaling. To date, little is known about the roles of α-terpineol produced by TPSs in plant defense. In *S. album*, the expression levels of genes coding for HMCR and FPPS were slightly induced in the leaves and stems of seedlings after the foliar application of 0.1% MeJA [46]. In our study, increased enzyme activities in the ROS antioxidant defense system and the sharp accumulation of *SaTPS1* and *SaTPS2* transcripts by exogenously applied MeJA and SA in leaves suggest that *SaTPS1* and *SaTPS2* play defensive roles against biotic stresses. Similarly, elevated levels of *SaTPS3* transcripts induced by MeJA in the three tissues (Fig. 10c) inferred that *SaTPS3* had similarly defensive roles to *SaTPS1* and *SaTPS2*. Moreover, MeJA led to increases of *SaTPS1* and *SaTPS2* transcripts in stems and roots (Fig. 10a and b). Collectively, this demonstrates that both local and systemic defense responses were triggered by exogenously applied MeJA in *S. album* seedlings. This result is the same as findings reported by Koo et al. (2009) [66] in Arabidopsis and Delaunois et al. (2014) [67] in grapevine, in which local activation of JA and its biologically active derivatives (i.e., MeJA) as JA signaling molecules spread systemically throughout the plant and induce JA responses in distant organs. In general, SA is mainly associated with resistance to biotrophic and hemibiotrophic pathogens, and triggers systemic acquired resistance [68, 69]. The reason why exogenous SA resulted in the downregulation of *SaTPS1* to *3* genes in treated roots needs to be further explored. Simultaneously, activated expression of *SaSSY* in leaves and stems by MeJA and SA offers a cue for further exploring the mechanism of santalol biosynthesis in *S. album*.

### *SaTPS1* to *3* play roles in response to abiotic stresses in different tissues

Environmental factors play important roles governing the emission of biogenic terpenes. The emission of some terpene volatiles is highly dependent on temperature, light and oxidative stresses [32, 38]. In loblolly pine (*Pinus taeda*), high light intensity and temperature induced the emission of (*E*)-β-farnesene and (*E*)-α-bergamotene [70]. Our previous work showed that *SaSS/BS* and *SaMonoTPS1* accumulated to high levels in the roots of *S. album* in response to 4°C [47]. In that study, the full-length of *SaSS/BS* was rediscovered as *SaTPS2* in this study. In the present study, the expression levels of *SaTPS2* in leaves and roots after exposure for 12 h to 4°C were similar to our previous results, in which a decrease of *SaTPS2* transcripts in leaves and significant up-regulation in roots were observed. Although adverse temperature and light inhibited the expression of *SaTPS1* to *3* genes in leaves, high or low temperature significantly induced an increase in *SaTPS1* to *3* transcripts in roots. High light intensity induced a remarkable elevation of *SaTPS1* and *SaTPS3* transcripts in stems and roots. There are two possible reasons for these trends. The first is that leaves are directly exposed to adverse stresses and responded more quickly to these stresses than stems and roots. The second is that there are differences in resource allocation between above- and underground tissues during the stress response. Nevertheless, these results imply that *SaTPS1* to *3* play a role in protecting sandalwood from abiotic stresses. In contrast, the decrease of almost all *SaSSY* transcripts in treated tissues suggests that environmental stresses affect the expression of *SaSSY*, unlike *SaTPS1* to *3*.

## Conclusions

After mining publicly-available RNA-seq data, three new terpene synthase genes were biochemically characterized. They were shown to be multifunctional mono- and sesqui-TPSs. The occurrence of functional divergence of TPS led to the formation of a diversity of terpenoids in *Santalum* spp. Our work demonstrated that *SaTPS1* to *3* genes play important roles in chemical defense and in protection against temperature and light stress. This study provides a basis for assessing the roles of SaTPSs in direct or indirect defense against pathogen attacks and in regulating environmental stresses. We are currently in the process of overexpressing *SaTPS1* to *3* as part of a program to genetically improve sandalwood for enhanced resistance to pathogens, such as powdery mildew, which severely infect *S. album* leaves [71]. Furthermore, greater attention should be paid to the environmental significance of the volatile terpenoids in *S. album* essential oil, which is present in the highly valued aromatic HW.

### Methods

### Plant material and stress treatment

Young leaves (YL) were harvested from 10-year-old mature *S. album* trees growing in a sandalwood plantation at the South China Botanical Garden. Wood shavings from SW and HW tissues were separately collected by drilling the stems of the same *S. album* trees at 30 cm from the ground using a Hagloff wood borer. To assess transcript accumulation of young trees, wood shavings from the stems of immature three-year-old trees (IW, immature wood) about 3 cm in diameter were also obtained. Tissue samples from three individuals were pooled as one biological replicate for gene expression and GC-MS analysis of volatiles, snap frozen in liquid nitrogen and stored at − 80°C until further use.

Culture of *S. album* seedlings followed our previous methods [47]. The foliage of six-month-old seedlings was sprayed at a rate of 10 ml per plant with 1 mM MeJA or SA solution which included 0.1% Tween 80 as a surfactant. The induction period was 24 h. Control plants were treated with a 0.1% Tween 80 solution at the same application dose. Treated plants were placed separately in different greenhouses in order to avoid cross-contamination under the same growth conditions. Seedlings with uniform growth were acclimated in a phytotron at 28°C/23°C (day/night), 14-h photoperiod, and 100 μmol m^− 2^ s^− 1^ photosynthetic photon flux density for one week. They were then exposed to 4°C and 38°C for 12 h, serving as low temperature or mild heat stress treatments, respectively. Plants grown under normal conditions were transferred to high light intensity (approx. 250 μmol m^− 2^ s^− 1^) for 12 h or to normal light as the control. Leaves, stems and roots were separately harvested after the completion of each treatment, quickly frozen in liquid nitrogen, and stored at − 80°C until use. Samples from three individuals were pooled as a biological replicate and repeated three times as three independent biological duplicates.

### Extraction of volatiles

Volatile compounds were extracted following the Celedon et al. (2016) [44] method with minor changes. Briefly, YL, SW, HW and IW samples were air dried for one week, extracted using 200 mg of ground tissue with 5 ml of pentane, spiked with *n*-dodecane as an internal standard (0.05 μl/ml), then mixed end-over-end for 48 h at room temperature. Samples were centrifuged at 2000 *g* at 4°C for 20 min and the pentane phase was transferred to a new GC vial. Then 1 μl was injected for GC/MS analysis after being reduced to 50 μl with a stream of dry nitrogen. Analyses were performed using three biological replicates, each of which comprised two technical replicates.

### Identification of terpene synthases in *S. album*

Raw reads were downloaded at BioProject IDs, PRJNA327296 for leaves [47], PRJNA297453 and SRR1725543 for stems [42, 44], and PRJNA243306 for roots [48] from the SRA database at NCBI. De novo transcriptome assembly was conducted using Trinity [72]. Finally, a single set of non-redundant unigenes were generated from all the unigenes using TGICL [73]. The TPS N-terminal domain (PF01397), TPS family metal binding domain (PF03936), prenyltransferase and squalene oxidase repeat (PF00432), and prenyltransferase like (PF13243) were downloaded from the Pfam database (http://pfam.xfam.org/), and used as bait to search against the *S. album* transcriptome with an E-value threshold of 10^− 5^.

### RNA extraction, gene isolation, and qRT-PCR

Total RNA was extracted according to Kolosova et al. (2004) [74] and Jones et al. (2011) [40]. Two micrograms of total RNA were reverse-transcribed using M-MLV reverse transcriptase according to the manufacturer’s instructions (Promega, Madison, WI, USA). Specific primers used to amplify full-length cDNAs are listed in Additional file 1: Table S8. The DNA molecules were amplified by PCR using high fidelity Platinum Taq DNA polymerase (Invitrogen), cloned into the pMD18-T vector (Takara Bio Inc., Dalian, China) and sequenced at the Beijing Genomics Institute (BGI). The gene-specific oligonucleotide primers used for qRT-PCR analysis are described in Additional file 1: Table S8. qRT-PCR was performed as described previously [47].

### Phylogenetic analysis

Multiple alignments were used by DNAMAN8.0 (Lynnon Biosoft, San Ramon, CA, USA) and a phylogenetic tree was constructed by the neighbour-joining (NJ) method using MEGA 6.0 [75].

### Subcellular localization of *SaTPS*s

For YFP fusion constructs, the coding regions of *SaTPS1* to *3* were subcloned into the pSAT6-EYFP vector using T4 ligase (Invitrogen) (Additional file 1: Table S8 and S9). All constructs were verified by DNA sequencing at BGI. The fusion constructs and m-Cherry fluorescence protein were transformed into Arabidopsis mesophyll protoplasts as described previously [76]. The transformed protoplasts were incubated at 22°C for 16–24 h. YFP fluorescence was observed using a confocal laser-scanning microscope (Leica TCS SP8 STED 3X, Wetzlar, Germany).

### Heterologous expression and purification of recombinant protein

Deletion of plastidial targeting peptide can improve the expression of functional mono-TPSs [77, 78]. For SaTPS1, two constructs were made, one with and one without the transit peptide. The *SaTPS* cDNA fragments obtained with primers designed with restriction enzyme sites at the ends were cloned into the pET-28a vector (Novagen) to generate 6His-SaTPSs (Additional file 1: Table S8 and S9). The constructs were verified by DNA sequencing at BGI. His-SaTPS recombinant proteins were induced into the *E. coli* Rossetta 2 (DE3) strain (Novagen) according to the method of Srivastava et al. [42]. Purification of the recombinant proteins was performed with Ni-NTA agarose (Qiagen) and the eluted proteins were desalted on a PD-10 desalting column (GE Healthcare) following the Jones et al. method [40]. The concentration of proteins was determined using the Bradford method [79]. The purified recombinant proteins were analyzed using 10% SDS-PAGE.

### In vitro enzyme assay

Enzymatic reactions were performed in a 2-ml screw-capped GC glass vial in a final volume of 500 μl containing 10 μg recombinant TPS, reaction buffer (25 mM HEPES, pH 7.4, 10 mM MgCl_2_ or MnCl_2_, 5 mM dithiothreitol, and 5% (v/v) glycerol), and 50 μM substrate (*E,E*)-FPP, GPP, or GGPP (Sigma-Aldrich). The reaction mixtures were carefully overlaid with 500 μl of *n*-hexane (Sigma-Aldrich) and incubated for 2 h at 30°C. The vial was then vigorously vortexed for 1 min to trap volatile products. After centrifuging at 2000 *g* and 4°C for 30 min, the upper hexane layer was transferred to a new 2-ml glass vial for GC-MS analysis. As negative controls, heat-inactivated recombinant protein and an empty vector were separately assayed.

### GC-MS analysis

GC-MS analysis was performed on a GCMS-QP2010 SE (Shimadzu Corporation, Kyoto, Japan) equipped with a HP-5MS column (30 m × 0.25 mm × 0.25 μm). The injector temperature was 230°C, splitless mode was used with a splitless time of 1 min, and helium was used as the carrier gas at a flow rate of 1.0 ml/min. The GC temperatures were 60°C for 3 min, ramp of 4°C/min to 230°C, and maintained at 230°C for 20 min. Scan ranges: 40–220 *m/z* for products of the in vitro enzyme assay and 20–500 *m/z* for compounds of volatiles. The volatile components and enzyme products were identified by comparison of their mass spectra with those in the NIST2005 (National Institute of Standards and Technology, Gaithersburg, MD, USA), NIST2005s, NIST2014, NIST2014s and FFNSC1.3 (Flavors and fragrances of natural and synthetic compounds version 1.3) library data for GC-MS and comparison of their retention indices (RIs). RIs were determined on the basis of an *n*-alkanes (C_8_-C_40_) mix standard (Sigma-Aldrich) under the same operation conditions. The identifications of authentic standards of six main products in the enzyme assays, including α-terpineol, linalool, geraniol, (*E*)-β-farnesene, (*E,E*)-nerolidol and (*E,E*)-farnesol were further verified by NMR (Fig. S11–28). Since no *(E)*-α-bergamotene and sesquisabinene standards were commercially available [80], their verifications were referred to those reported by Srivastava et al. (2015) [42].

### Steady-state kinetics

Estimates of the *V*_*max*_ and *K*_*m*_ of SaTPS1, SaTPS2 and SaTPS3 were determined by using the malachite green phosphate assay kit (Sigma-Aldrich, No. MAK307) following the method of Vardakou et al. (2014) [81]. One microgram of proteins with varying concentrations (0.5 μM to 50 μM) of substrates FPP or GPP and Mg^2+^ in 500 μl of 25 mM HEPES buffer was reacted at 30°C for 10 min. The reaction was terminated by adding 10 μl of 5 M HCl. Released free phosphate was determined as described in the assay kit manual. The data was fit to a nonlinear regression of the Michaelis-Menten equation by using GraphPad Prism 7 to obtain *V*_*max*_ and *K*_*m*_.

### Antioxidant enzyme assays

The activities of antioxidant enzymes, including SOD (EC1.15.1.1), CAT (EC1.11.1.6), POD (EC1.11.1.7) and APX (EC1.11.1.11) of leaves, stems and roots of untreated and treated samples were separately tested using activity assay kits (product codes BC0175 for SOD, BC0200 for CAT, BC0095 for POD, and BC0220 for APX; Solarbio, Beijing, China). A total of 100 mg of plant tissue was crushed using a mortar and pestle in corresponding extraction buffer and the supernatant was collected after centrifuging at 8000 or 13,000 *g* for 20 min at 4°C. SOD activity was determined on the basis of the inhibition of the photochemical reduction of nitro blue tetrazolium at 560 nm [82]. CAT activity was expressed as 1 nM of H_2_O_2_ degradation/min at 240 nm. One unit of POD activity was defined as the increase by one unit/min at 470 nm. APX activity was expressed as 1 μM of ascorbate oxidized/min at 290 nm.

## Additional file

Additional file 1: **Table S1.** Composition of volatiles from four sandalwood tissues. **Table S2.** Length distribution of assembled transcripts and unigenes. **Table S3.** Terpene synthase identified based on transcriptome data. **Table S4.** Information of three SaTPSs isolated from *S. album.* **Table S5.** TPS proteins from other plant species used in phylogenetic analysis. **Table S6.** Predicted chloroplast transit peptides. **Table S7.** In vitro assay products that each recombination SaTPS and FPP or GPP. **Table S8.** List of primers used in this study. **Table S9** Restriction enzymes used for YPF plasmid construction and expression vectors. **Figure S1.** Length distribution of *S. album* unigenes. **Figure S2.** Agarose gel electrophoresis of three *SaTPS* ORFs. **Figure S3.** Comparison of deduced amino acid sequences of SaTPS1 and two other TPSs. **Figure S4.** Comparison of transcript levels of *SaTPS*s. **Figure S5.** SDS-PAGE analysis of recombinant proteins. **Figure S6.** In vitro enzymatic assays of recombinant SaTPS1 using GPP and Mn^2+^. **Figure S7.** In vitro enzyme assays of recombinant SaTPS2 using FPP and Mn^2+^. **Figure S8.** In vitro enzyme assays of recombinant SaTPS2 using GPP and Mn^2+^. **Figure S9.** In vitro enzymatic assays of recombinant SaTPS3 using GPP and Mn^2+^. **Figure S10.** Michaelis-Menten plots for three SaTPSs. **Figure S11–28.** Characterization of six authentic standards by NMR. (PDF 2014 kb)

## Abbreviations

APX: Ascorbate peroxidase
BS: Bisabolene synthase
CAT: Catalase
CYP: Cytochrome P450
FPP: Farnesyl diphosphate
FPPS: Farnesyl diphosphate synthase
GGPP: Geranylgeranyl diphosphate
GPP: Geranyl diphosphate
HMCR: Hydroxymethylglutaryl CoA reductase
HW: Heartwood
IW: Immature wood
JA: Jasmonic acid
MeJA: Methyl jasmonate
POD: Peroxidase
RI: Retention index
ROS: Reactive oxygen species
SA: Salicylic acid
SOD: Superoxide dismutase
SQS1: sesquisabinene synthase 1
SQS2: sesquisabinene synthase 2
SRC: Sequence Read Archive
SS/BS: Santalene/bergamotene synthase 1
SSY: Santalene synthase
SW: Sapwood
Tp: Transit peptide
TPS: Terpene synthase
YFP: Yellow fluorescent protein
YL: Young leaf
