# Supplementary material for: Identification and functional characterization of three new terpene synthase genes involved in chemical defense and abiotic stresses in Santalum album
Source: BMC Plant Biol. 2019 Mar 28;19:115. doi: 10.1186/s12870-019-1720-3 (PMC6437863; doi:10.1186/s12870-019-1720-3)
Supplement: Supplementary file 1 — Table S1. Composition of volatiles from four sandalwood tissues. Table S2. Length distribution of assembled transcripts and unigenes. Table S3. Terpene synthase identified based on transcriptome data. Table S4. Information of three SaTPSs isolated from S. album. Table S5. TPS proteins from other plant species used in phylogenetic analysis. Table S6. Predicted chloroplast transit peptides. Table S7. In vitro assay products that each recombination SaTPS and FPP or GPP. Table S8. List of primers used in this study. Table S9 Restriction enzymes used for YPF plasmid construction and expression vectors. Figure S1. Length distribution of S. album unigenes. Figure S2. Agarose gel electrophoresis of three SaTPS ORFs. Figure S3. Comparison of deduced amino acid sequences of SaTPS1 and two other TPSs. Figure S4. Comparison of transcript levels of SaTPSs. Figure S5. SDS-PAGE analysis of recombinant proteins. Figure S6. In vitro enzymatic assays of recombinant SaTPS1 using GPP and Mn2+. Figure S7. In vitro enzyme assays of recombinant SaTPS2 using FPP and Mn2+. Figure S8. In vitro enzyme assays of recombinant SaTPS2 using GPP and Mn2+. Figure S9. In vitro enzymatic assays of recombinant SaTPS3 using GPP and Mn2+. Figure S10. Michaelis-Menten plots for three SaTPSs. Figure S11–28. Characterization of six authentic standards by NMR. (PDF 2014 kb) [file 12870_2019_1720_MOESM1_ESM.pdf]

Supplementary information

Identification and functional characterization of three new terpene synthase genes involved in chemical defense and abiotic stresses in *Santalum album*

Xinhua Zhang[1#*], Meiyun Niu[1,2#], Jaime A. Teixeira da Silva[3], Yueya Zhang[1,2], Yunfei Yuan[1], Yongxia Jia[1], Yangyang Xiao[1], Yuan Li[1], Lin Fang[1], Songjun Zeng[1], Guohua Ma[1]

[1]Key Laboratory of South China Agricultural Plant Molecular Analysis and Genetic Improvement, South China Botanical Garden, Chinese Academy of Sciences, Guangzhou, China

[2]University of the Chinese Academy of Sciences, Beijing, China

[3]P. O. Box 7, Miki cho post office, Ikenobe 3011-2, Kagawa-Ken, 761-0799, Japan

[*]Corresponding author, xhzhang@scib.ac.cn

[#]These authors equally contributed to this work.

Table S1. Composition of volatiles from four sandalwood tissues

Peak No.[a]	Compounds	RT	RI	Lit. RI	Percent total (relative %)			
					YL	IW	SW	HW
1	α-santalene	18.49	1409	1422	18.3	-	-	5.0
2	(*E*)-α-bergamotene	18.88	1424	1430	2.0	-	-	-
3	*epi*-β-santalene	19.18	1436	1450	19.6	-	-	3.8
4	(*E*)-β-farnesene	19.35	1443	1445	1.5	-	-	-
5	β-santalene	19.50	1449	1470	23.2	-	-	5.5
6	β-bisabolene	20.68	1496	1503	4.9	-	-	-
7	unknown	20.93	1506	-	-	-	-	2.6
8	α-santalol	24.65	1666	1683	27.3	58.2	51.8	45.2
9	α-*trans*-bergamotol	24.95	1680	1708	-	6.2	5.4	6.2
10	α-santalol isomer	25.06	1685	-	-	-	-	0.7
11	*epi*-β-santalol	25.22	1692	1721	-	2.5	1.8	2.7
12	unknown	25.38	1699	-	-	1.7	1.5	0.6
13	β-santalol	25.54	1706	1720	3.2	24.4	19.4	24.3
14	β-santalol isomer	25.98	1727	-	-	1.9	1.9	1.2
15	lanceol	26.44	1748	1744	-	5.1	18.2	2.2

[a]: Order of elution

RT: retention time (min)

RI: retention indices calculated against C_8-C_{40} *n*-alkanes on the HP-5MS column

Lit. RI: retention data in literature

YL, young leaves, IW, immature wood, SW, sapwood, HW, heartwood

Table S2. Length distribution of assembled transcripts and unigenes

Nucleotide length (bp)	Transcripts			Unigenes			All unigenes
	L	S	R	L	S	R	
300-500	29959	63433	30628	23255	50659	22807	49302
500-1000	35673	64940	30551	16291	44608	13739	45830
1000-2000	68170	51337	36990	9020	19516	8751	26816
2000+	133751	93535	29977	7492	18589	5198	42600
Total number	299278	273245	163163	84139	133372	80808	164548
Total length	6.05×10^8	5.65×10^8	1.89×10^8	6.44×10^7	1.63×10^8	5.34×10^7	2.16×10^8
N50 length	2996	3926	1968	1366	2267	1081	2562
Mean length	2023.131	2066.05	1159.968	764.9966	1219.76	660.2269	1311.39

L: leaves, S: stems, R: roots

Table S3. Terpene synthase identified based on transcriptome data

GeneID	Unigenes length (bp)	Pfam_IDs	Nr annotation
**Core terpene synthases**			
SaCL18358Contig1	2388	PF03936.11;PF01397.16	Full=Santalene synthase; Short=SaSSy;
SaCL35655Contig1	2311	PF03936.11;PF01397.16;PF06749.7	sesquisabinene B synthase 1 [*Santalum album*]
SaCL8704Contig1	2134	PF03936.11;PF01397.16	Full=(+)-α-terpineol synthase; Short=SaMonoTPS1 [*Santalum album*]
SaCL1444Contig1	3319	PF03936.11;PF01397.16	hypothetical protein CISIN 1g011444mg [*Citrus sinensis*]
SaCL1444Contig2	3429	PF03936.11;PF01397.16	hypothetical protein EUGRSUZ_D00677 [*Eucalyptus grandis*]
SaCL3724Contig1	3120	PF03936.11	ent-kaurene synthase [*Castanea mollissima*]
SaCL35155Contig1	2237	PF03936.11	copalyl diphosphate synthase [*Castanea mollissima*]
SaCL15826Contig1	1049	PF03936.11	PREDICTED: (3S,6E)-nerolidol synthase 1, chloroplastic-like isoform X1 [*Vitis vinifera*]
SaCL22168Contig1	425	PF01397.16	unnamed protein product [*Vitis vinifera*]
SaCL24607Contig1	425	PF03936.11	hypothetical protein JCGZ_00771 [*Jatropha curcas*]
SaCL30007Contig1	574	PF03936.11	unnamed protein product [*Vitis vinifera*]
SaCL34136Contig1	378	PF01397.16	hypothetical protein PRUPE_ppa003380mg [*Prunus persica*]
L_Unigene46835	2118	PF03936.11;PF01397.16	PREDICTED: (-)-α-terpineol synthase [*Vitis vinifera*]
L_Unigene24940	2041	PF03936.11;PF01397.16	santalene bergamotene synthase 1 [*Santalum album*]
L_Unigene23790	2065	PF03936.11;PF01397.16	Full=β-bisabolene synthase; Short=SauBS [*Santalum austrocaledonicum*]
L_Unigene47029	760	PF01397.16	(3S)-linalool/(*E*)-nerolidol synthase [*Vitis vinifera*]
L_Unigene52373	689	PF01397.16	terpene synthase [*Camellia sinensis*]
L_Unigene57844	377	PF03936.11	unnamed protein product [*Vitis vinifera*]
L_Unigene59759	459	PF03936.11	hypothetical protein EUGRSUZ_K00827 [*Eucalyptus grandis*]
L_Unigene73773	404	PF01397.16	Probable terpene synthase 12 GN=TPS12 OS=*Ricinus communis* (castor bean) PE=3 SV=1
L_Unigene63397	418	PF01397.16	Full=Probable sesquiterpene synthase; Short=SspiSTPS [*Santalum spicatum*]
L_Unigene57499	887	PF03936.11;PF01397.16	Full=Probable sesquiterpene synthase; Short=SaSTPS [*Santalum album*]
L_Unigene35622	2040	PF01397.16;PF03936.11	E,E-α-farnesene synthase, putative [*Theobroma cacao*]
S_Unigene50447	293	PF01397.16	Probable terpene synthase 13 GN=TPS13 OS=*Ricinus communis* (castor bean) PE=3 SV=1
S _Unigene54662	458	PF03936.11	PREDICTED: probable terpene synthase 9 [*Prunus mume*]
S _Unigene18428	1952	PF01397.16	sesquisabinene B synthase 2 [*Santalum album*]
S _Unigene43351	298	PF01397.16	santalene bergamotene synthase 1 [*Santalum album*]
S _Unigene1341	574	PF03936.11	sesquisabinene B synthase 2 [*Santalum album*]
R_Unigene43572	543	PF03936.11	Full=Monoterpene synthase; Short=SaMonoTPS1 [*Santalum album*]
R_Unigene43573	973	PF01397.16;PF01397.16	sesquisabinene B synthase 2 [*Santalum album*]
R_Unigene23324	2161	PF01397.16;PF03936.11	(-)-germacrene D synthase [*Vitis vinifera*]
R_Unigene48444	2824	PF03936.11;PF01397.16;PF06749.7	PREDICTED: probable terpene synthase 9 [*Vitis vinifera*]
R_Unigene17444	592	PF03936.11	(E,E)-α-farnesene synthase GN=AFS1 OS=*Malus domestica* (apple) PE=1 SV=2
R_Unigene2927	421	PF01397.16	geraniol synthase [*Vitis vinifera*]
R_Unigene5000	611	PF03936.11	geraniol synthase, partial [*Rosa rugosa*]
R_Unigene67165	472	PF01397.16	unnamed protein product [*Vitis vinifera*]
R_Unigene67440	263	PF03936.11	1,8-cineole synthase, chloroplast precursor, putative [*Ricinus communis*]
R_Unigene71544	501	PF03936.11	PREDICTED: (3S,6E)-nerolidol synthase 1-like isoform X1 [*Pyrus* x *bretschneideri*]
**Triterpene-specific synthases**			
SaCL25648Contig1	2827	PF13243.1;PF13249.1;PF00432.16 PF13243.1;PF13249.1;PF09492.5	PREDICTED: lupeol synthase isoform X1 [*Vitis vinifera*]
SaCL29604Contig1	2663	PF00432.16;PF13249.1;PF13243.1;PF09492.5	β-amyrin synthase [*Aralia elata*]
SaCL7379Contig1	3649	PF00432.16;PF13249.1	hypothetical protein PRUPE_ppa022710mg [*Prunus persica*]
SaCL811Contig1	2917	PF00432.16;PF13249.1	cycloartenol synthase protein [*Azadirachta indica*]
SaCL811Contig3	1338	PF00432.16;PF13249.1;PF13243.1;PF09492.5	triterpene synthase [*Eugenia uniflora*]
L_Unigene_37502	3625	PF13243.1;PF13249.1;PF00432.16 PF13243.1;PF13249.1;PF09492.5	RecName: Full=β-amyrin synthase [*Betula platyphylla*]
L_Unigene_77770	384	PF13243.1;PF00432.16	putative lanosterol synthase [*Erysiphe necator*]
S _Unigene_13498	1209	PF00432.16;PF13249.1;PF13243.1;PF09492.5	hypothetical protein PRUPE_ppa022710mg [*Prunus persica*]
R_Unigene_28839	306	PF13249.1;PF00432.16;PF13243.1	cycloartenol synthase [*Medicago truncatula*]

L_Unigene, S _Unigene or R_Unigene indicate that Unigenes were specific to leaves (L), stems (S) or roots (R).

Table S4. Information of three SaTPSs isolated from *S. album*

**Candidate transcripts**	**Protein**	**CDS sequence length (bp)**	**Protein sequence length (aa)**	**PI**	**MW**	**cTP**
Unigene46835	SaTPS1	1815	604	6.01	69.26	Y
Unigene24940	SaTPS2	1695	564	5.02	64.58	-
Unigene23790	SaTPS3	1713	570	5.03	65.55	-

PI, isoelectric point; MW, molecular weight; cTP, chloroplast transit peptide

Table S5. TPS proteins from other plant species used in phylogenetic analysis

**Species**	**TPS**	**Accession ID in NCBI**
Santalum album	monoterpene synthase	ACF24767
S. album	β-bisabolene synthase	AIV42941
S. austrocaledonicum	β-bisabolene synthase	ADO87003
S. spicatum	α-bisabololsynthase	E3W206
S. album	sesquisabinene B synthase 1	AIV42939
S. album	sesquisabinene B synthase 2	AIV42940
S. spicatum	sesquisabinene B synthase	KM091272
S. album	santalene synthase	ADO87000
S. austrocaledonicum	santalene synthase	ADO87001
S. spicatum	santalene synthase	ADO87002
Vitis vinifera	(-)-α-terpineol synthase	AAS79352
Arabidopsis thaliana	β-caryophyllene/α-humulene synthase	AAO85539
Cucumis sativus	(*E*,*E*)-α-farnesene synthase	Q66PX9
S. austrocaledonicum	sesquiterpene synthase	E3W207
S. album	sesquiterpene synthase	ACF24768
S. spicatum	sesquiterpene synthase	E3W208
A. thaliana	S-(+)-linalool synthase	Q84UV0
Antirrhinum majus	nerolidol/linalool synthase 1	ABR24417
V. vinifera	(3S)-linalool/(*E*)-nerolidol synthase	ADR74212
Camellia sinensis	(*E*)-nerolidol synthase	KY033151
Pinus abies	(*E*)-α-bisabolene synthase	Q675L6
Abies grandis	(*E*)-α-bisabolene synthase	AAC24192
P. taeda	α-farnesene synthase	Q84KL5
P. abies	(-)-linalool synthase	Q675L2
P. taeda	(-)-α-terpineol synthase	Q84KL4
Solanum lycopersicum	copalyl diphosphate synthase	BAA84918
Oryza sativa	*ent*-copalyl diphosphate synthase 1	Q6ET36
S. lycopersicum	*ent*-kaurene synthase	AEP82778
O. sativa	kaurene synthase 1	Q0JA82
S. habrochaites	santalene and bergamotene synthase	ACJ38409
Clarkia breweri	linalool synthase 2	AAD19840
V. vinifera	P(*E*)-nerolidol/(*E*,*E*)-geranyl linalool synthase	NP001268004
Actinidia deliciosa	terpene synthase	ACO40485

Table S6. Predicted chloroplast transit peptides

Name	Length	Score	cTP	CS-score	cTP-length
SaTPS1	604	0.567	Y	4.261	49
SaMonoTPS	576	0.469	-	5.213	25
V. vinifera α-terpineol synthase	627	0.540	Y	3.414	52

The prediction cTP/no cTP is based solely on this score. cTP, chloroplast transit peptide. CS-score is the MEME scoring matrix score for the suggested cleavage site.

Table S7. *In vitro* assays products that each recombine SaTPS and FPP or GPP

Enzyme	Substrate	Products	RT	RI	Lit. RI	Total (relative %)	
						Mg^{2+}	Mn^{2+}
SaTPS1	GPP	α-thujene	5.15	900	904	0.7	0.3
		α-pinene	5.20	906	935	6.0	5.5
		sabinene	5.83	951	974	14.9	5.1
		β-pinene	5.89	955	988	1.4	1.2
		myrcene	6.08	969	992	10.8	5.7
		limonene	6.66	1009	1024	1.0	5.5
		cineole	6.69	1011	1036	1.8	0.7
		β-ocimene	6.91	1027	1038	1.1	1.6
		linalool	7.60	1076	1100	11.7	35.8
		α-terpineol	8.78	1170	1193	45.7	25.7
		geraniol	9.45	1227	1224	4.9	12.9
SaTPS2	FPP	7-*epi*-sesquithujene	10.93	1367	1381	3.2	-
		unknown	11.07	1381	-	1.5	7.0
		α-bergamotene isomer	11.20	1394	-	2.5	6.2
		(*E*)-α-bergamotene	11.40	1415	1430	24.8	22.4
		(*E*)-β-farnesene	11.51	1428	1445	1.5	-
		sesquisabinene	11.58	1436	1444	33.0	35.6
		unknown	11.86	1468	-	2.2	1.3
		α-zingiberene	11.90	1472	1489	3.1	6.2
		α-bisabolene	11.95	1478	1494	0.9	0.7
		β-bisabolene	12.02	1486	1503	9.0	6.3
		γ-bisabolene	12.09	1494	1525	1.6	1.2
		unknown	12.14	1501	-	4.9	1.6
		γ-bisabolene isomer	12.21	1509	-	7.9	11.5
		β-bisabolol	13.18	1644	1675	1.9	-
		α-bisabolol	13.25	1654	1699	2.0	-
	GPP	α-thujene	5.15	900	904	1.9	3.5
		α-pinene	5.19	905	935	2.6	7.8
		sabinene	5.81	950	974	0.5	4.2
		β-pinene	5.87	954	988	1.0	-
		myrcene	6.09	969	992	5.2	6.3
		limonene	6.68	1011	1024	4.8	6.1
		linalool	7.61	1077	1100	64.9	64.8
		α-terpineol	8.79	1171	1193	11.1	4.7
		geraniol	9.46	1228	1224	8.0	2.6
SaTPS3	FPP	cedrene	11.25	1399	1409	3.5	-
		unknown	11.37	1412	-	13.8	-
		(*E*)-β-farnesene	11.49	1425	1445	20.7	-
		unknown	11.94	1476	-	3.4	-
		γ-bisabolene	12.07	1492	1525	13.8	-
		(*E*)-nerolidol	12.37	1531	1561	29.8	-
		(*E,E*)-farnesol	13.47	1685	1667	21.3	-
	GPP	myrcene	6.09	969	992	10.2	8.3
		limonene	6.67	1010	1024	2.8	5.8
		unknown	6.77	1017	-	3.2	-
		β-ocimene	6.92	1028	1041	3.8	11.9
		linalool	7.60	1077	1100	48.8	53.3
		α-terpineol	8.78	1170	1193	4.6	6.5
		geraniol	9.46	1228	1224	26.6	14.2

RT: retention time (min)

RI; retention indices calculated against C_8-C_{40} *n*-alkanes on the HP-5MS column

Lit. RI: retention data in literature

Table S8. List of primers used in this study

Primer name	Sequence (5′-3′)
SaTPS1 ORF	Forward-5′- ATGGCTTTTGGTGCTATTCCTAG-3′
	Reverse-5′-TCAGCAACTCGGAAAAGACTCAAT-3′
SaTPS2 ORF	Forward-5′-ATGGATTCAGCCACCCTAAAGGTTC-3′
	Reverse-5′-TCAGTTTAAGACTCGTCGTCAACT-3′
SaTPS3 ORF	Forward-5′-ATGGAGTCTTTTGTTTTTCTGAAGT-3′
	Reverse-5′-ATTCAGTCTTCATCGAGTGGGATT-3′
pSAT6-EYFP-N1:	Forward-5′-CGCGTCGACGGATGGCTTTTGGTGCTATTCCTAG-3′
SaTPS1	Reverse-5′-CGCGGATCCCGCAACTCGGAAAAGACTCAAT-3′
pSAT6-EYFP-N1:	Forward-5′-CCGCTCGAGCATGGATTCAGCCACCCTAAAGGTTC-3′
SaTPS2	Reverse-5′-CGCGGATCCCAGACTCGTCGTCAACTGAAATCGGA-3′
pSAT6-EYFP-N1:	Forward-5′-GGGAAGCTTCATGGAGTCTTTTGTTTTTCT-3′
SaTPS3	Reverse-5′-GCGGTCGACTGTCTTCATCGAGTGGGATTG-3′
pET28a: SaTPS1	Forward-5′-GAGGGATCCATGGTCCGGAGATCCGCCAACTA-3′
	Reverse-5′-GAGGCGGCCGCAACTCGGAAAAGACTCAAT-3′
pET28a: SaTPS2	Forward-5′-GAGGGATCCATGGATTCAGCCACCCTAAAGGTTC-3′
	Reverse-5′-GAGGCGGCCGCTTAAGACTCGTCGTCAACTGAAATC-3′
pET28a: SaTPS3	Forward-5′-GCGTCGACAAATGGAGTCTTTTGTTTTTCTG-3′
	Reverse-5′-GAGCTCGAGTCAGTCTTCATCGAGTGGGATTG-3′
SaTPS1 QRT	Forward-5′-CTTACCTGGTAGAGGCAAAGTG-3′
	Reverse-5′-CCAGAATCAGTGGAGCAGATATT-3′
SaTPS2 QRT	Forward-5′-CGAGGGTGCACTGGATAAA-3′
	Reverse-5′-CGTCCGAAGAGGTTGCTAAA-3′
SaTPS3 QRT	Forward-5′- AAGCCGAGAATGGAGGAATAC-3′
	Reverse-5′-GTCTACCGTTTCCTTGCTTAGA-3′
SaSSY QRT	Forward-5′-CCTTCCTGATCTTCTGCACTAC-3′
	Reverse-5′-ATTATCGCCTCTTGCCATCTC-3′
SaActin QRT	Forward-5′-GTCACACGGTGCCAATCTAT-3′
	Reverse-5′-TACCCTCTCTCAGTCAGAATCTT-3′

Table S9. Restriction enzymes used YPF plasmid construction and expression vectors

**Name**	**Restriction site used for cloning**	**Expression vector**	**Expression cells**
SaTPS1	*Sal*I at 5' and *Bam*HI at 3'	pSAT6-EYFP-N1	Arabidopsis mesophyll protoplasts
SaTPS2	*Sal*I at 5' and *Hind*III at 3'		
SaTPS3	*Xho*I at 5' and *Bam*HI at 3'		
SaTPS1	*Bam*HI at 5' and *Not*I at 3'	pET28a	Rosetta 2 (DE3)
SaTPS2	*Bam*HI at 5' and *Not*I at 3'		
SaTPS3	*Hind*III at 5' and *Xho*I at 3'		

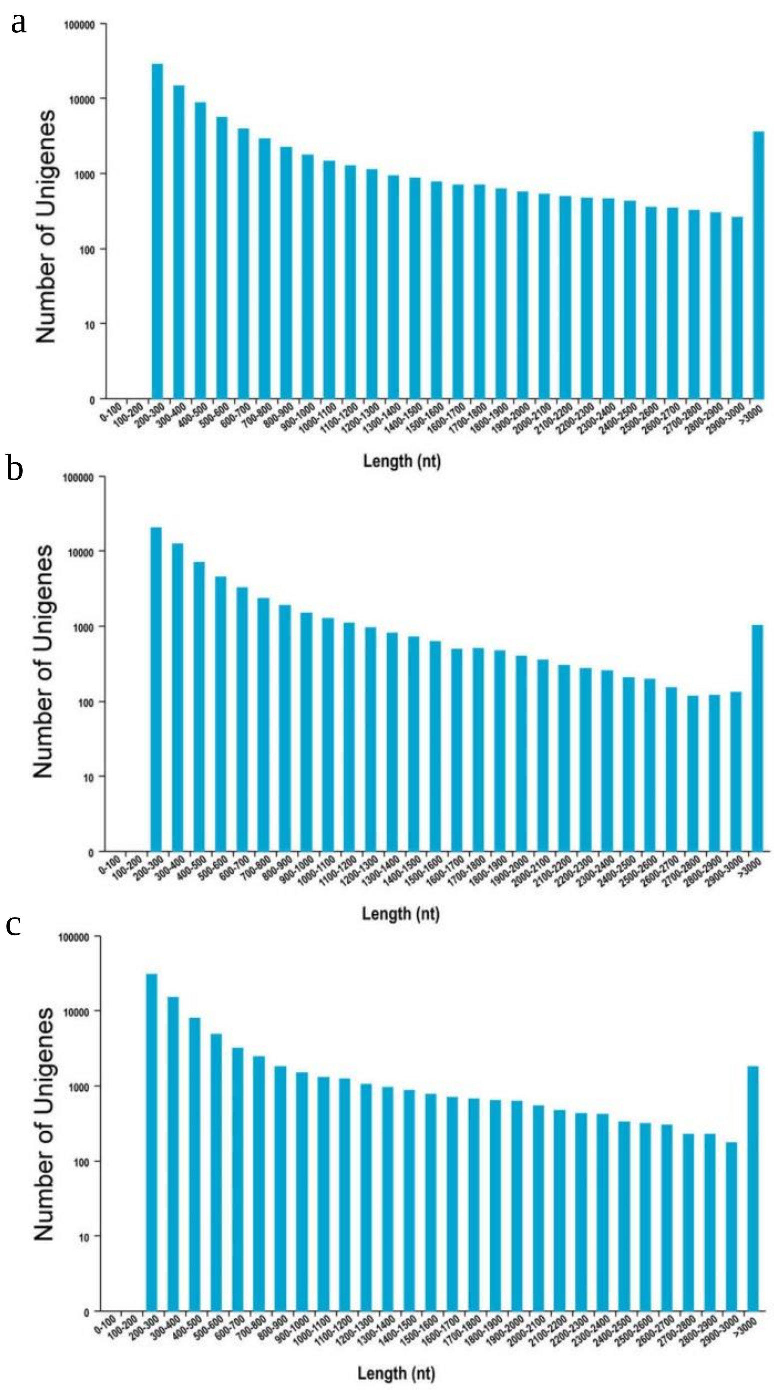

Fig. S1. Length distribution of *S. album* unigenes. a, leaves; b, roots; c, stems.

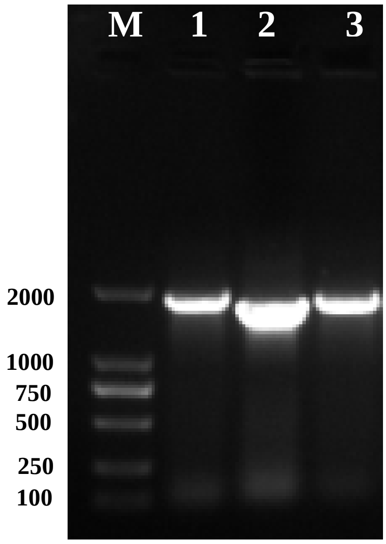

Fig. S2. Agarose gel electrophoresis of three *SaTPS*s ORFs. Lane 1: *SaTPS1*; lane 2: *SaTPS2*; lane 3: *SaTPS3*; M: DNA marker = 2000 kb.

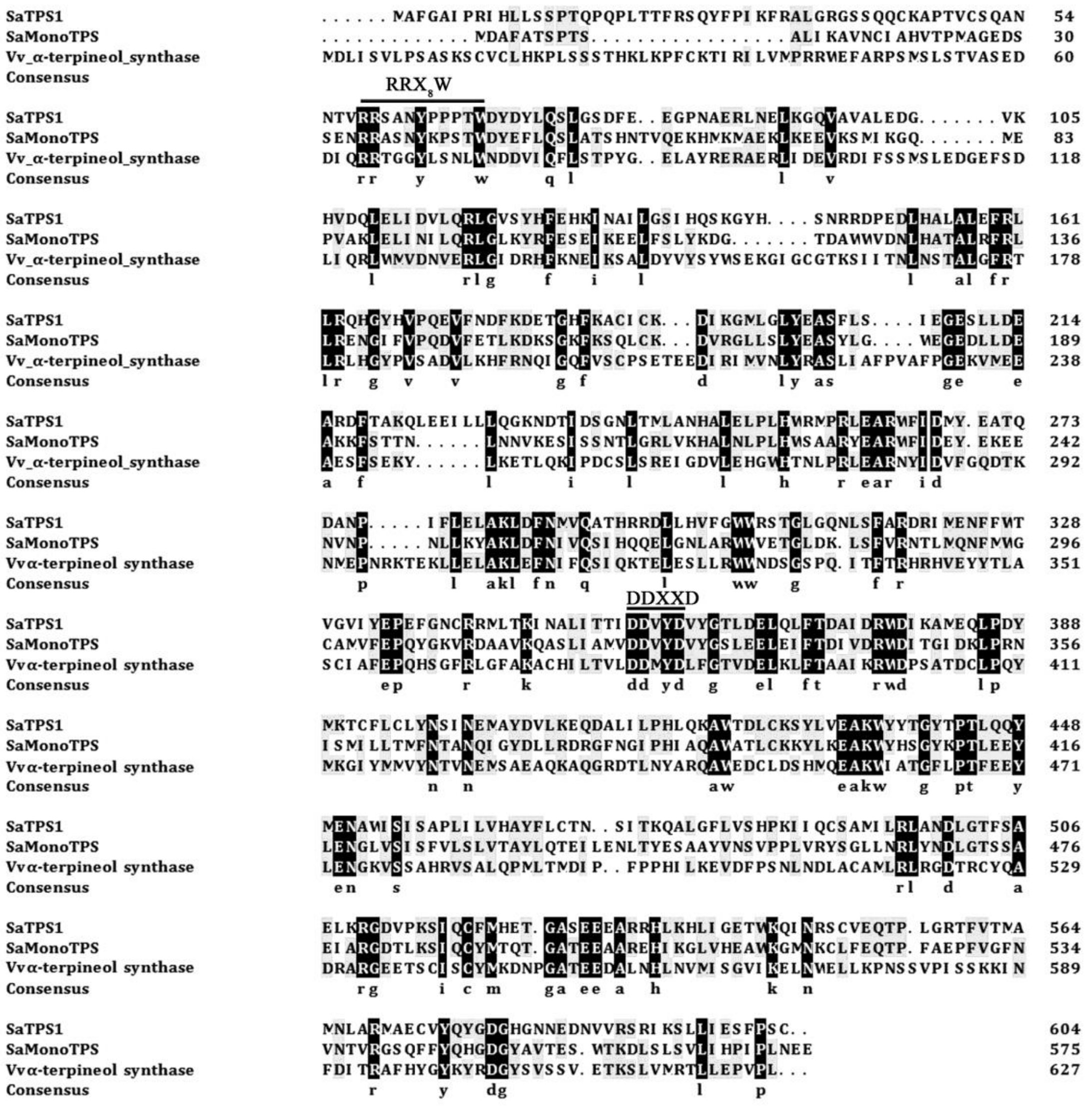

Fig. S3. Comparison of deduced amino acid sequences of SaTPS1 and two other TPSs. Conserved regions known for RRX_8W and DDXXD motifs are shown. Completely conserved residues are shaded in dark and similar residues are shaded in light gray. Dashes indicate gaps introduced to maximize sequence alignment.

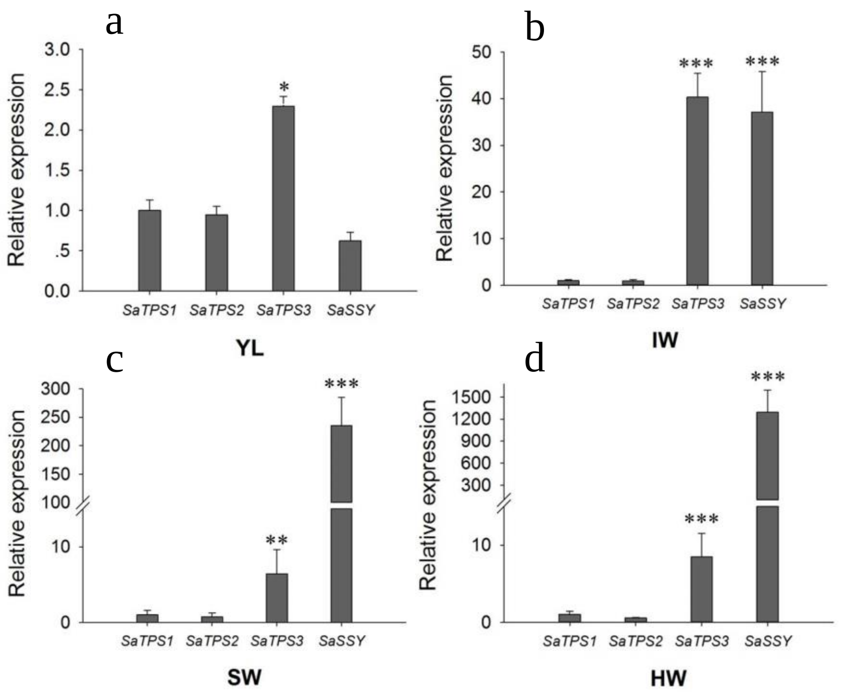

Fig. S4. Comparison of transcript levels of *SaTPSs*. a, YL, young leaves; b, IW, immature wood; c, SW, sapwood; d, HW, heartwood. Measurements were averaged from the results of three replicated experiments and statistically treated using a *t*-test. *$P < 0.05$, **$P < 0.01$ and ***$P < 0.001$.

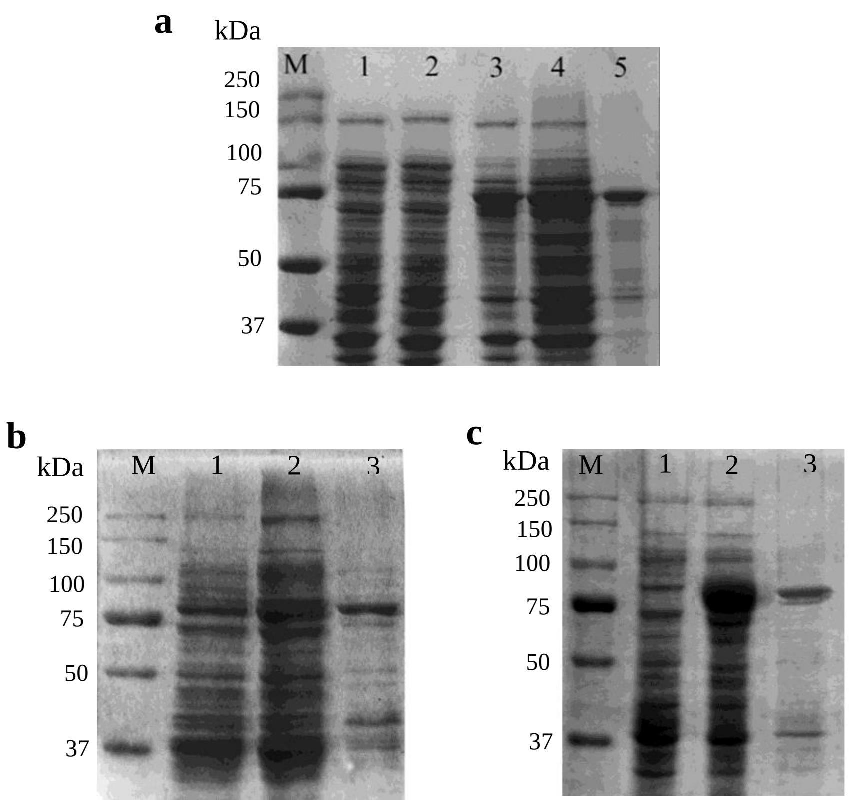

Fig. S5. SDS-PAGE analysis of recombinant proteins. (a), Lane 1: supernatant of crude extracts from *E. coli* cells with expression of empty vector pET28a; Lane 2: supernatant of crude extracts from *E. coli* cells with expression of empty vector pET28a:SaTPS1 with the transit peptide; Lane 3: supernatant of crude extracts from *E. coli* cells with expression of empty vector pET28a:SaTPS1 without the transit peptide after being induced for 12 h; Lane 4: supernatant of crude extracts from *E. coli* cells with expression of empty vector pET28a:SaTPS1 without the transit peptide after being induced for 24 h; Land 5, purified recombinant SaTPS1. (b), Lane 1: supernatant of crude extracts from *E. coli* cells with expression of empty vector pET28a; Lane 2: supernatant of crude extracts from *E. coli* cells with expression of empty vector pET28a:SaTPS2; Lane 3: purified recombinant SaTPS2. (c), Lane 1: supernatant of crude extracts from *E. coli* cells with expression of empty vector pET28a; Lane 2: supernatant of crude extracts from *E. coli* cells with expression of empty vector pET28a:SaTPS3; Lane 3: purified recombinant SaTPS3. M, protein marker, which was showed in a, b, and c. kDa: kilodalton.

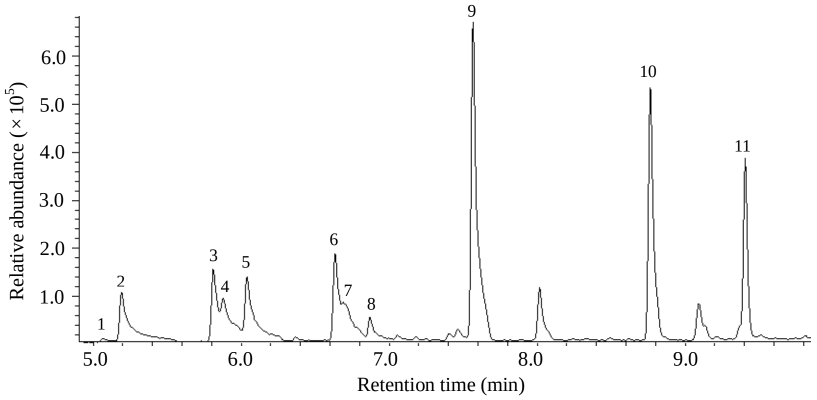

Fig. S6. *In vitro* enzymatic assays of recombinant SaTPS1 using GPP and Mn^{2+}. The reaction products were analyzed by GC-MS. Peaks marked with numbers were identified by mass spectra reference libraries NIST2005, NIST2005s, NIST2014, NIST2014s and FFNSC1.3 and comparison of their retention indices. Peaks: 1, α-thujene, 2, α-pinene, 3, sabinene, 4, β-pinene, 5, myrcene, 6, limonene, 7, cineole, 8, β-ocimene, 9, linalool, 10, α-terpineol, 11, geraniol.

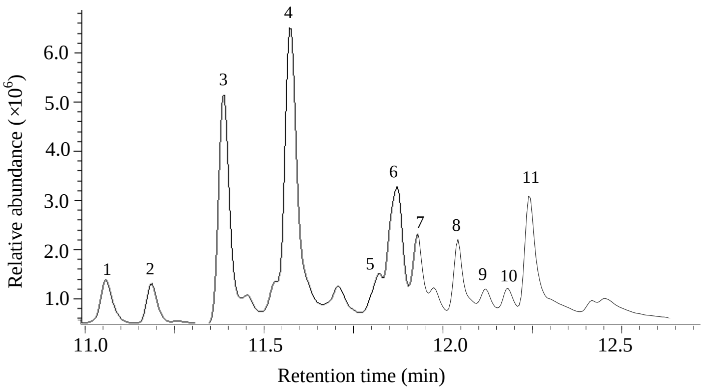

Fig. S7. *In vitro* enzyme assays of recombinant SaTPS2 using FPP and Mn^{2+}. The reaction products were analyzed by GC-MS. Peaks marked with numbers were identified by mass spectra reference libraries NIST2005, NIST2005s, NIST2014, NIST2014s and FFNSC1.3 and comparison of their retention indices. Peaks: 1, unknown, 2, α-bergamotene isomer, 3, (*E*)-α-bergamotene, 4, sesquisabinene, 5, unknown, 6, α-zingiberene, 7, α-bisabolene, 8, β-bisabolene, 9, γ-bisabolene, 10, unknown, 11, γ-bisabolene isomer.

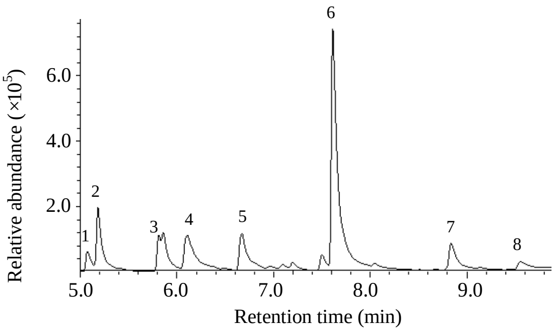

Fig. S8. *In vitro* enzyme assays of recombinant SaTPS2 using GPP and Mn^{2+}. The reaction products were analyzed by GC-MS. The peaks marked with numbers were identified by the mass spectra reference libraries NIST2005, NIST2005s, NIST2014, NIST2014s and FFNSC1.3 and comparison of their retention indices. Peaks: 1, α-thujene, 2, α-pinene, 3, sabinene, 4, myrcene, 5, limonene, 6, linalool, 7, α-terpineol, 8, geraniol.

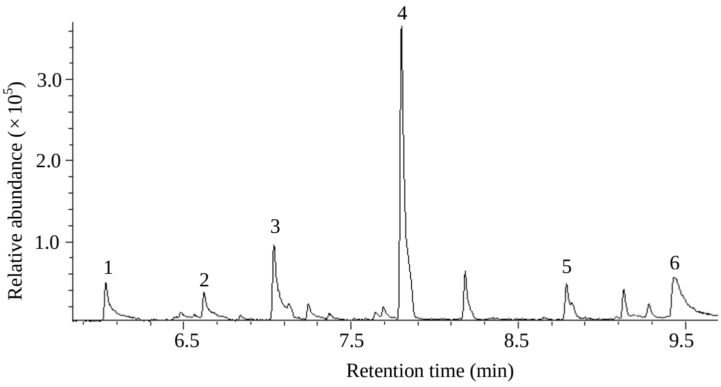

Fig. S9. *In vitro* enzymatic assays of recombinant SaTPS3 using GPP and Mn^{2+}. The reaction products were analyzed by GC-MS. The peaks marked with numbers were identified by the mass spectra reference libraries NIST2005, NIST2005s, NIST2014, NIST2014s and FFNSC1.3 and comparison of their retention indices. Peaks: 1, myrcene, 2, limonene, 3, β-ocimene, 4, linalool, 5, α-terpineol, 6, geraniol.

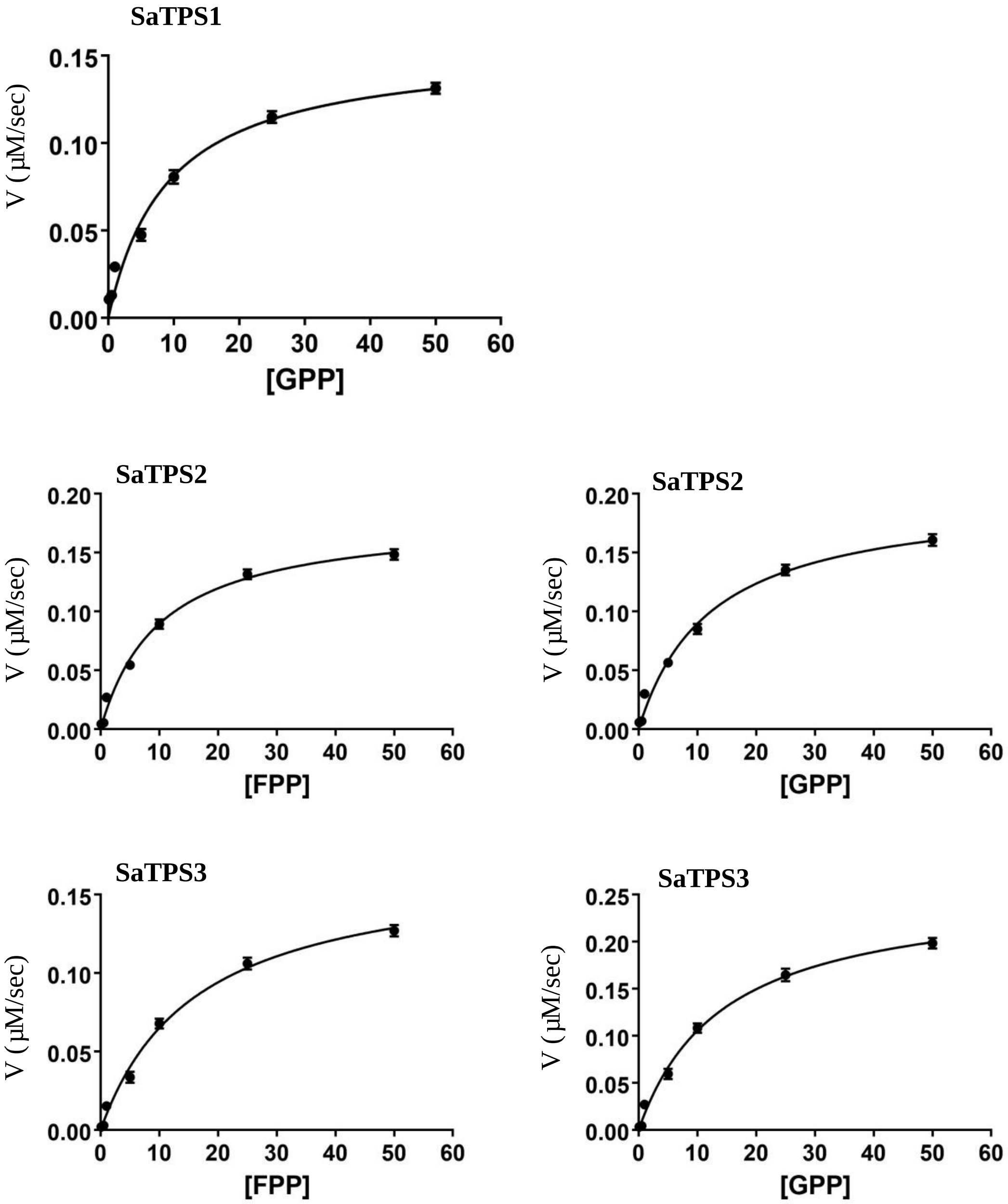

Fig. S10. Michaelis-Menten plots for three SaTPSs. Each data point was determined from triplicate experiments.

Spectral data for characterization of authentic standards

α-Terpineol: ^{1}H NMR (500 MHz, $CDCl_3$): δ 5.36 (m, 1H), 1.84-1.88 (m, 1H), 1.73-1.78 (m, 2H), 1.63 (s, 3H), 1.44-1.51 (m, 1H), 1.21-1.27 (m, 3H), 1.17 (s, 3H), 1.15 (s, 3H); ^{13}C NMR (125 MHz, $CDCl_3$): δ 134.0 (=C), 120.5 (=CH), 72.8 (-C-OH), 45.0 (-CH-), 31.0 ($-CH_2$), 27.4 ($-CH_3$), 26.9 ($-CH_2$), 26.2 ($-CH_3$), 23.9 ($-CH_2$), 23.3 ($-CH_3$); GC-EI-MS (70 eV): *m/z*: 136.0 [M^+], 121.0, 107.0, 93.0, 81.0, 67.0, 59.0 (100%), 55.0, 43.0.

Geraniol: ^{1}H NMR (500 MHz, $CDCl_3$): δ 5.37-5.40 (m, 1H), 5.05-5.08 (m, 1H), 4.13 (d, *J* = 6.96 Hz, 2H), 1.99-2.08 (m, 4H), 1.65 (s, 6H), 1.58 (s, 3H); ^{13}C NMR (125 MHz, $CDCl_3$) δ 139.8 (=C), 131.7 (=C), 123.9 (=CH), 123.3 (=CH), 59.4 ($-CH_2OH$), 39.5 ($-CH_2$), 26.3 ($-CH_2$), 25.6 ($-CH_3$), 17.7 ($-CH_3$), 16.2 ($-CH_3$); GC-EI-MS (70 eV): *m/z*: 154.0 [M^+], 139.0, 123.0, 111.0, 107.0, 93.0, 84.0, 69.0 (100%), 53.0, 41.0.

Linalool: ^{1}H NMR (500 MHz, CDCl3) 7: δ 5.89 (dd, *J* = 10.7, 17.5 Hz, 1H), 5.20 (dd, *J* = 1.01, 17.5 Hz, 1H), 5.08-5.11 (m, 1H), 5.04 (dd, *J* = 10.7, 1.01 Hz, 1H), 1.96-2.02 (m, 2H), 1.66 (s, 3H), 1.62 (s, 3H), 1.51-1.58 (m, 2H), 1.25 (s, 3H); ^{13}C NMR (125 MHz, $CDCl_3$): δ 145.0 (=CH), 132.0 (=C), 124.3 (=CH), 111.7 ($=CH_2$), 73.5 (-C-OH), 42.0 ($-CH_2$), 27.9 ($-CH_3$), 25.7 ($-CH_3$), 22.8 ($-CH_2$), 17.7 ($-CH_3$); GC-EI-MS (70 eV): *m/z*: 136.0 [M^+], 121.0, 107.0, 93.0, 80.0, 71.0 (100%), 55.0, 41.0.

(*E*)-β-Farnesene: ^{1}H NMR (500 MHz, $CDCl_3$): δ 6.36 (dd, *J* = 10.1,16.9Hz, 1H), 5.23 (d, *J* = 16.9Hz, 1H), 5.14 (m, 1H), 5.08(m, 1H), 5.04(d, *J* = 10.1 Hz, 1H), 5.00 (s, 1H), 4.98 (s, 1H), 2.14-2.24 (m, 4H), 2.02-2.08 (m, 2H), 1.94-1.99 (m, 2H) 1.66 (s, 3H), 1.58 (s, 6H); ^{13}C NMR (125 MHz, CDCl3) δ 146.1 (=C), 138.9 (=CH), 135.4 (=C), 131.3 (=C), 124.4 (=CH), 124.0 (=CH), 115.7 ($=CH_2$), 113.0 ($=CH_2$), 40.2 (-C-), 39.7 ($-CH_2$), 31.4($-CH_2$), 26.7 ($-CH_2$), 26.6 ($-CH_2$), 25.7 ($-CH_3$),17.7 ($-CH_3$),16.0($-CH_3$); GC-EI-MS (70 eV): m/z: 204.2 [M+], 189.0, 161.0, 147.0, 133.0, 120.0, 107.0, 93.0, 79.0, 69.0 (100%), 55.0, 41.0.

(*E*)-Nerolidol: ^{1}H NMR (500 MHz, $CDCl_3$): δ 5.89 (dd, *J* = 7.34, 17.03 Hz, 1H), 5.19 (dd, *J* = 1.16, 17.03 Hz, 1H), 5.11 (m, 1H), 5.06 (m, 1H), 5.04 (dd, *J* = 1.16, 7.34 Hz, 1H), 2.01-2.04 (m, 2H), 1.94-1.97 (m, 2H), 1.53-1.59 (m, 2H), 1.23-1.31 (m, 2H), 1.65 (s, 3H) 1.57 (s, 6H), 1.26 (s, 3H); ^{13}C NMR (125 MHz, $CDCl_3$) δ 145.1 (=CH), 135.6 (=C), 131.4 (=C), 124.3 (=CH), 124.2 (=CH), 111.7 ($=CH_2$), 73.5 (-C-), 42.1 ($-CH_2$), 39.7 ($-CH_2$), 27.9 ($-CH_3$), 26.6 ($-CH_2$), 25.7 ($-CH_3$), 22.7 ($-CH_2$), 17.7 ($-CH_3$), 16.0 ($-CH_2$); GC-EI-MS (70 eV): *m/z*: 204.0 [M^+], 189.0, 161.0, 136.0, 121.0, 107.0, 93.0, 81.0, 69.0 (100%), 55.0, 41.0.

(*E*,*E*)-Farnesol: ^{1}H NMR (500 MHz, $CDCl_3$): δ 5.38-5.41 (m, 1H), 5.05-5.10 (m, 2H), 4.13 (d, *J* = 6.96 Hz, 2H), 1.95-2.08 (m, 8H), 1.66 (s, 6H), 1.58 (s, 6H); ^{13}C NMR (125 MHz, $CDCl_3$) δ 139.8 (=C), 135.4 (=C), 131.3 (=C), 124.3 (=CH), 123.7 (=CH), 123.3 (=CH), 59.4 ($-CH_2OH$), 39.7 ($-CH_2$), 39.5 ($-CH_2$), 26.7 ($-CH_2$), 26.3 ($-CH_2$), 25.7 ($-CH_3$), 17.7 ($-CH_3$), 16.3 (CH_3), 16.0 (CH_3); GC-EI-MS (70 eV): *m/z*: 191.0 [M^+], 161.0, 136.0, 121.0, 107.0, 95.0, 93.0, 81.0, 69.0 (100%), 55.0, 41.0.

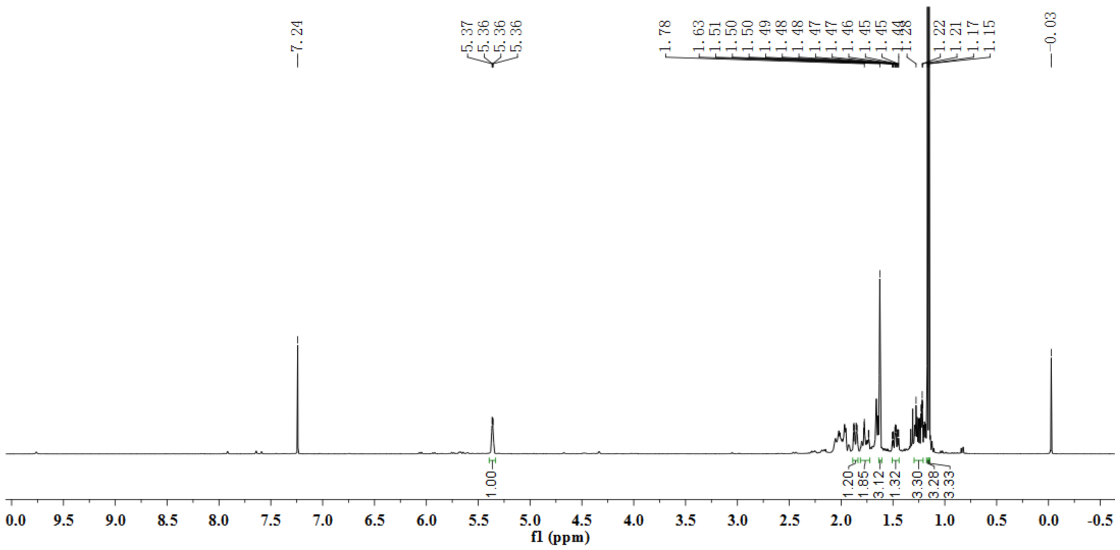

Fig S 11. ^{1}H NMR of α-terpineol in $CDCl_3$ at 500 MHz.

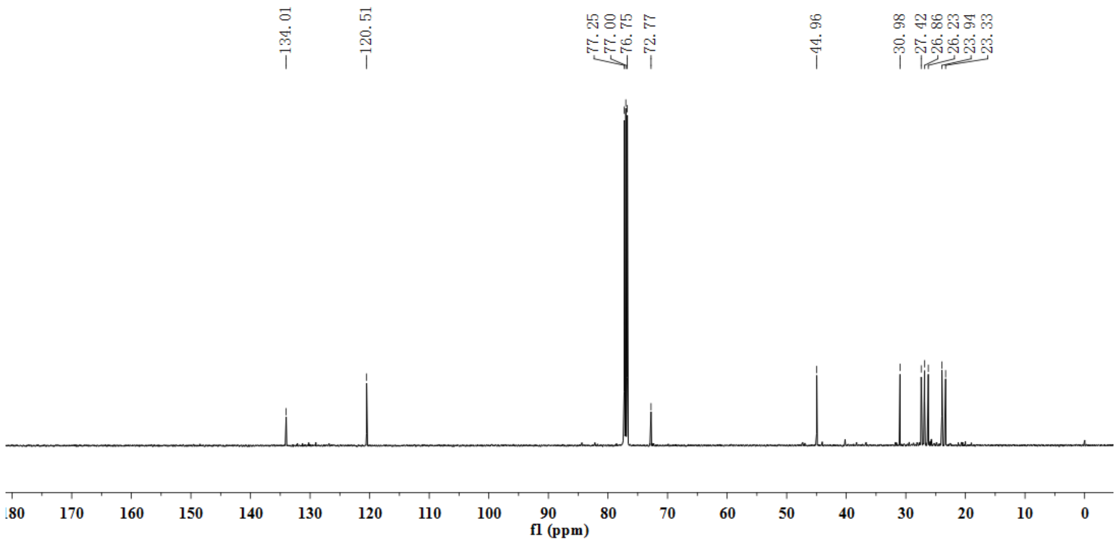

Fig S 12. ^{13}C NMR of α-terpineol in $CDCl_3$ at 125 MHz.

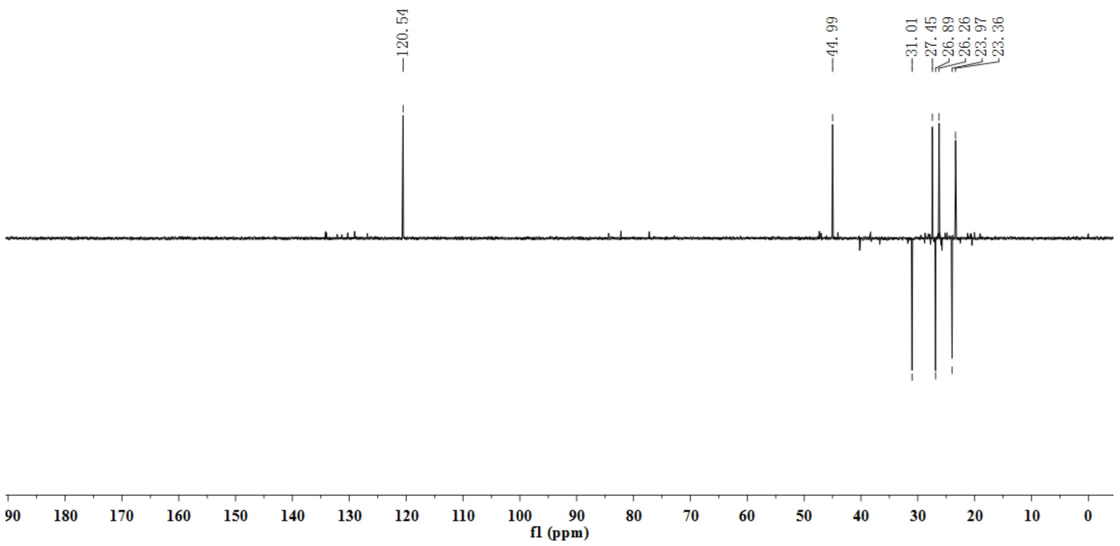

Fig S 13. DEPT NMR of α-terpineol in $CDCl_3$ at 125 MHz.

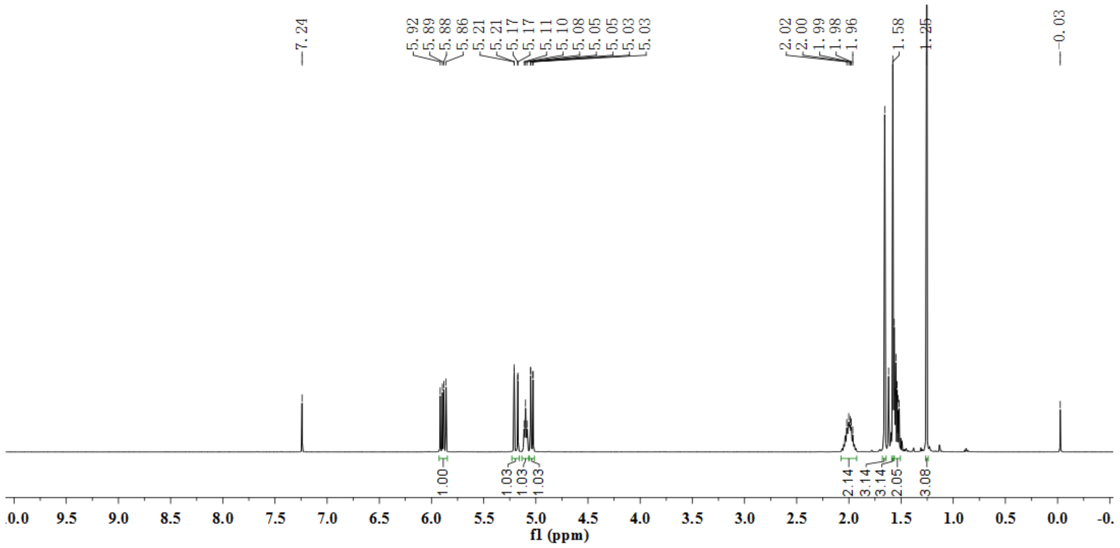

Fig S 14. ^{1}H NMR of linalool in $CDCl_3$ at 500 MHz.

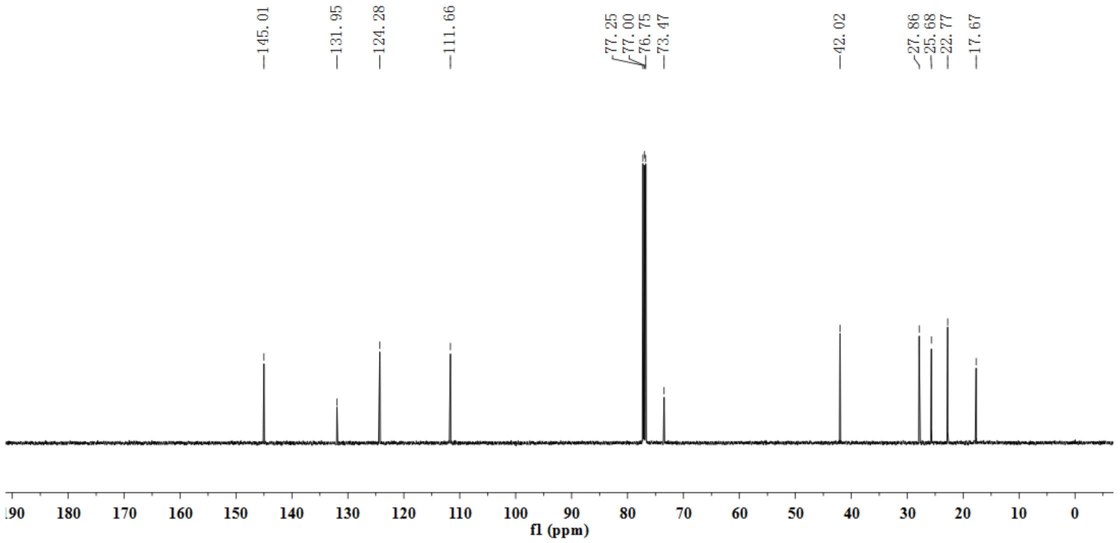

Fig S 15. ^{13}C NMR of linalool in $CDCl_3$ at 125 MHz.

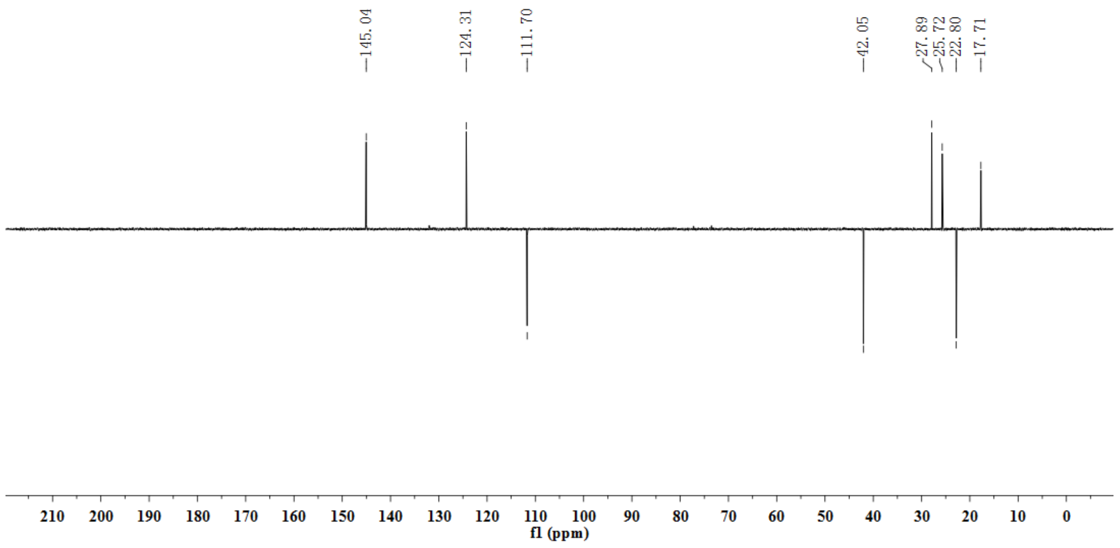

Fig S 16. DEPT NMR of linalool in $CDCl_3$ at 125 MHz.

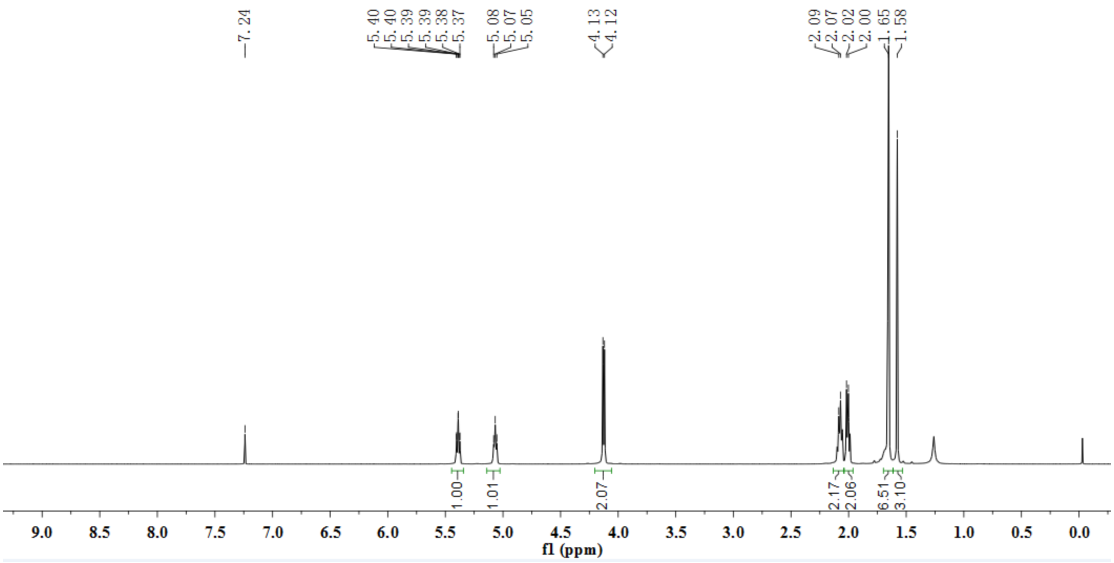

Fig S 17. ^{1}H NMR of geraniol in $CDCl_3$ at 500 MHz.

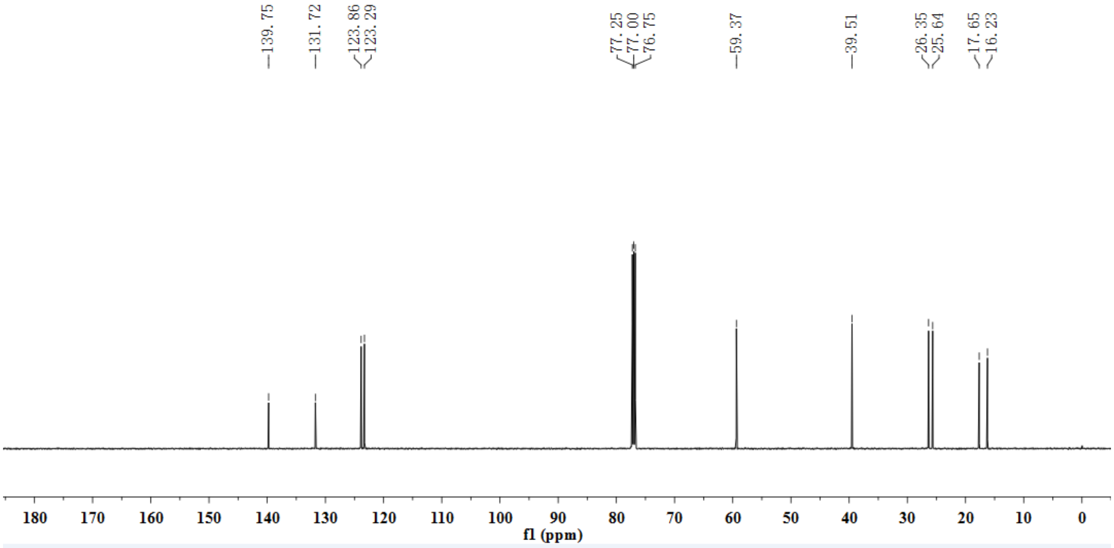

Fig S 18. ^{13}C NMR of geraniol in $CDCl_3$ at 125 MHz.

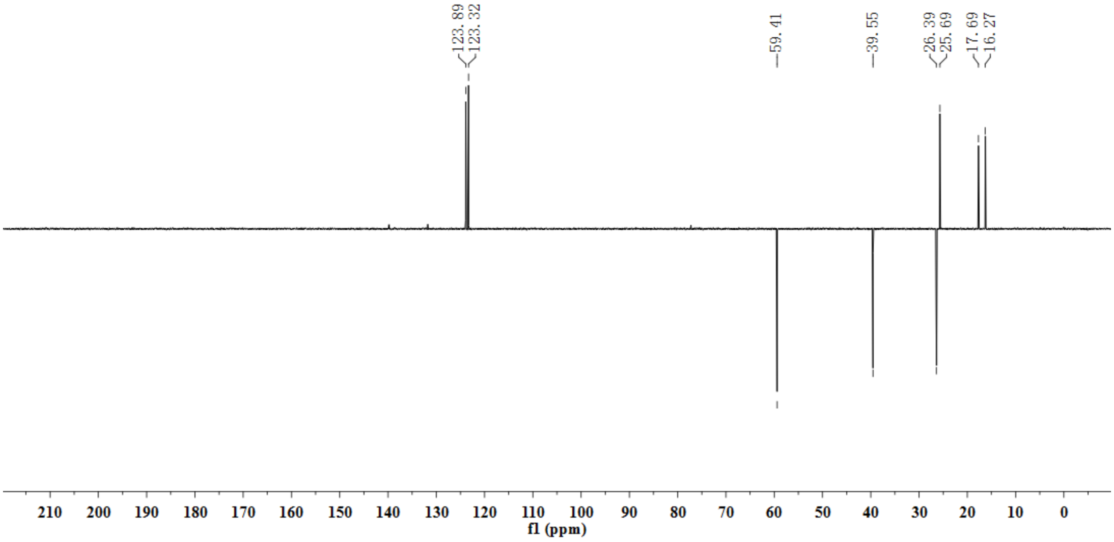

Fig S 19. DEPT NMR of geraniol in $CDCl_3$ at 125 MHz.

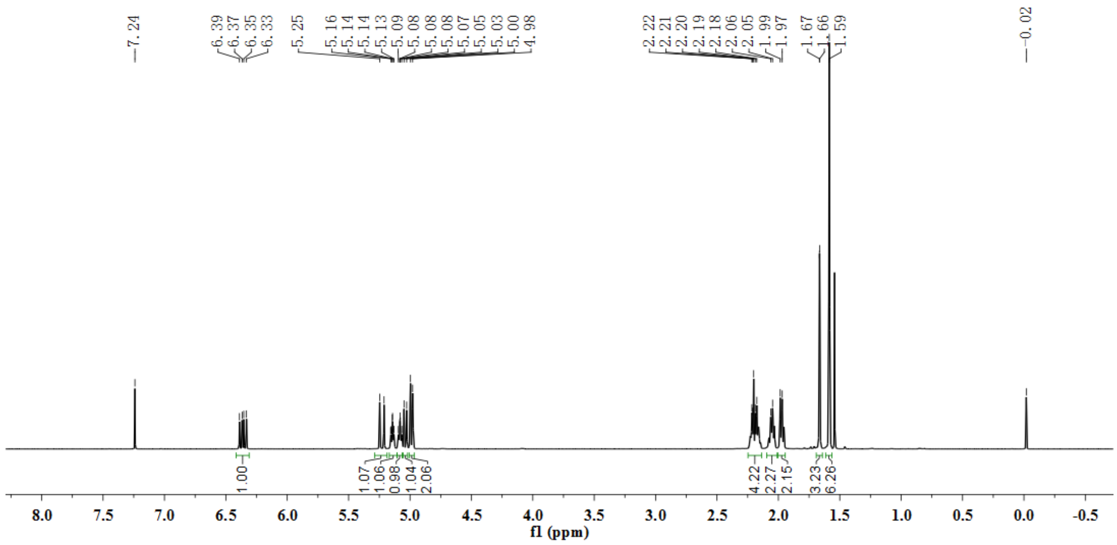

Fig S20. ^{1}H NMR of (*E*)-β-farnesene in $CDCl_3$ at 500 MHz.

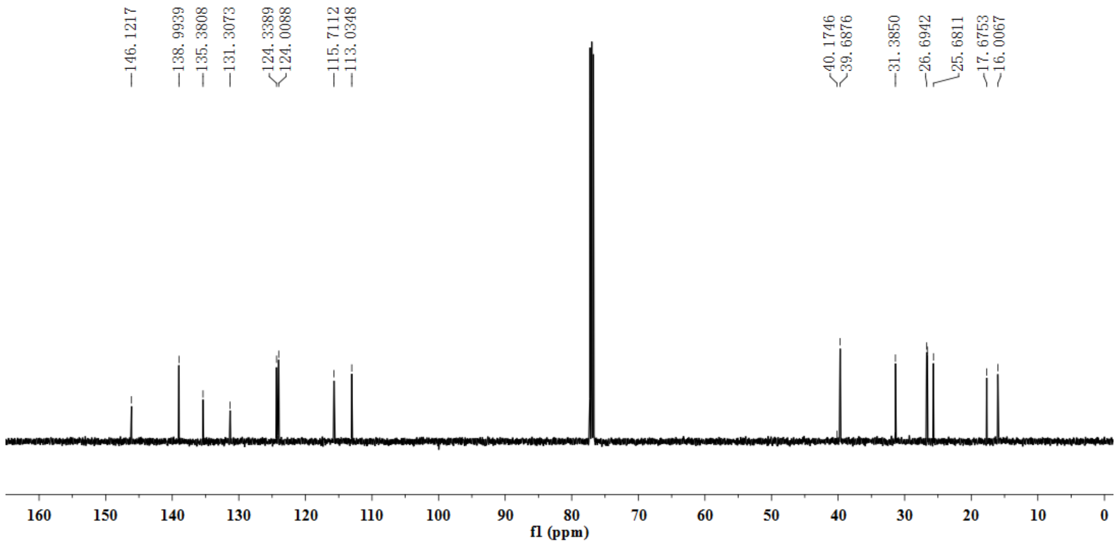

Fig S21. ^{13}C NMR of (*E*)-β-farnesene in $CDCl_3$ at 125 MHz.

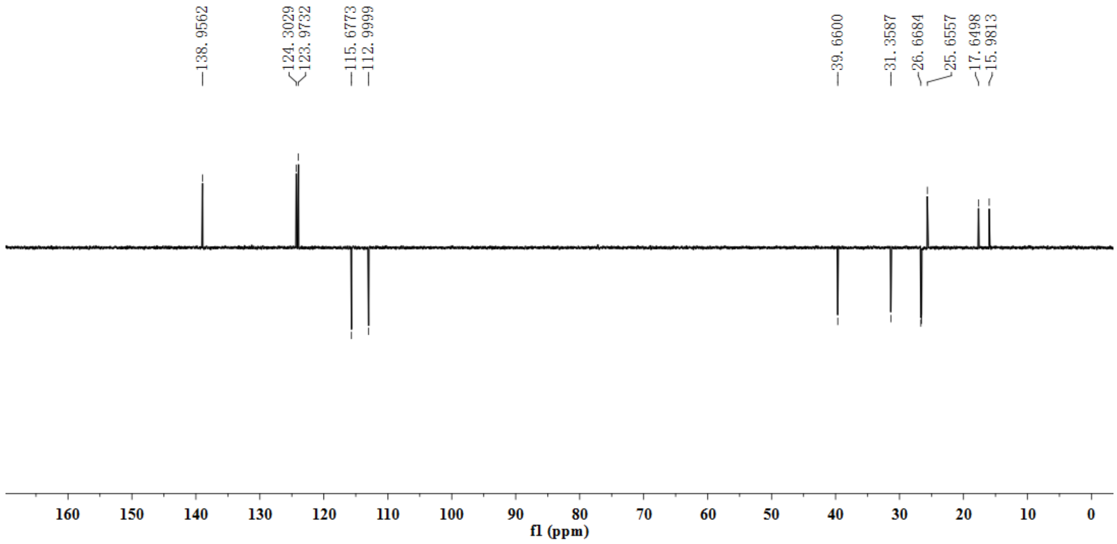

Fig S22. DEPT NMR of (*E*)-β-farnesene in $CDCl_3$ at 125 MHz.

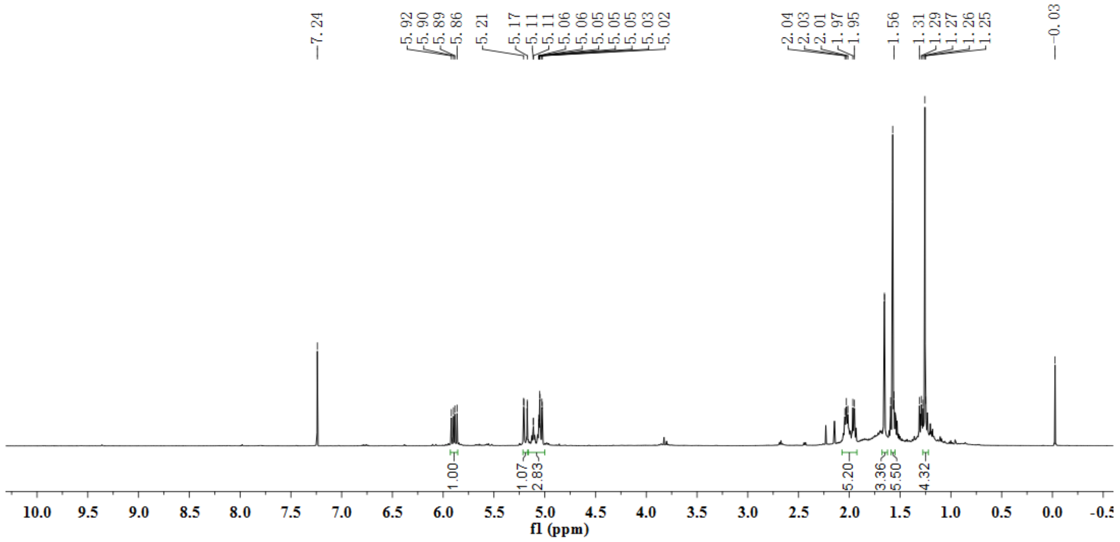

Fig S23. ^{1}H NMR of (*E*)-nerolidol in $CDCl_3$ at 500 MHz.

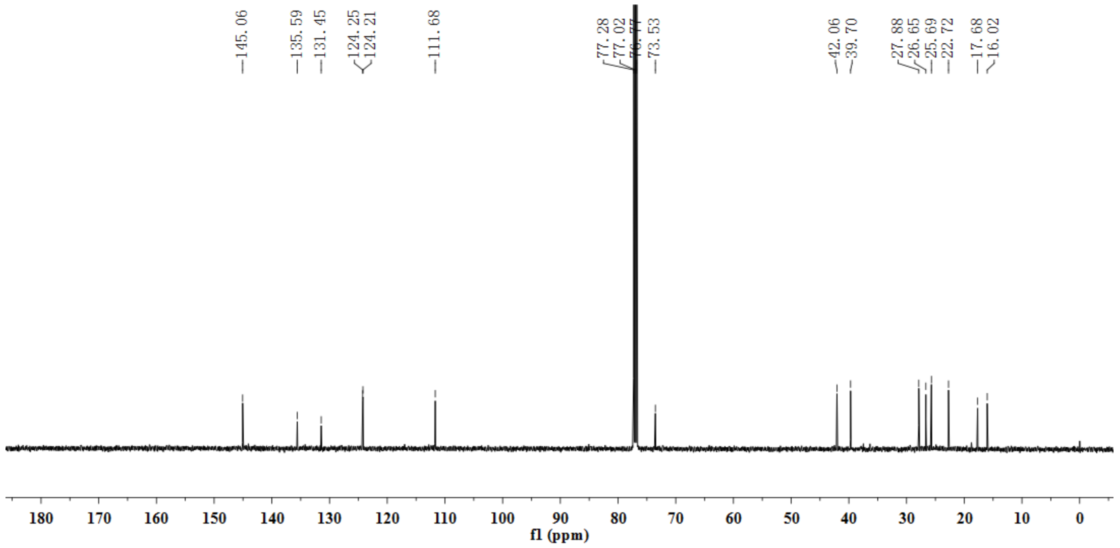

Fig S24. ^{13}C NMR of (*E*)-nerolidol in $CDCl_3$ at 125 MHz.

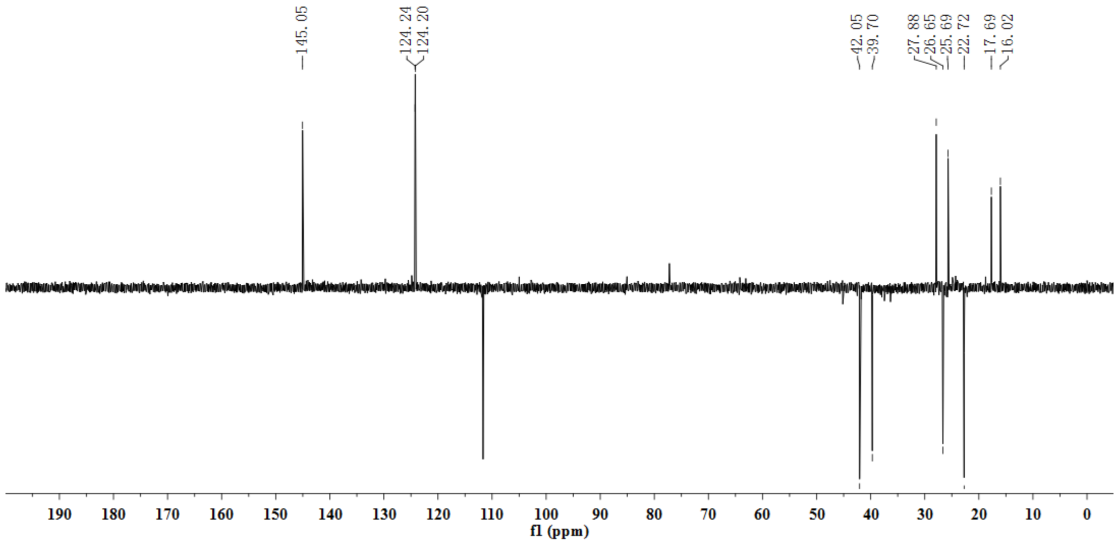

Fig S25. DEPT NMR of (*E*)-nerolidol in $CDCl_3$ at 125 MHz.

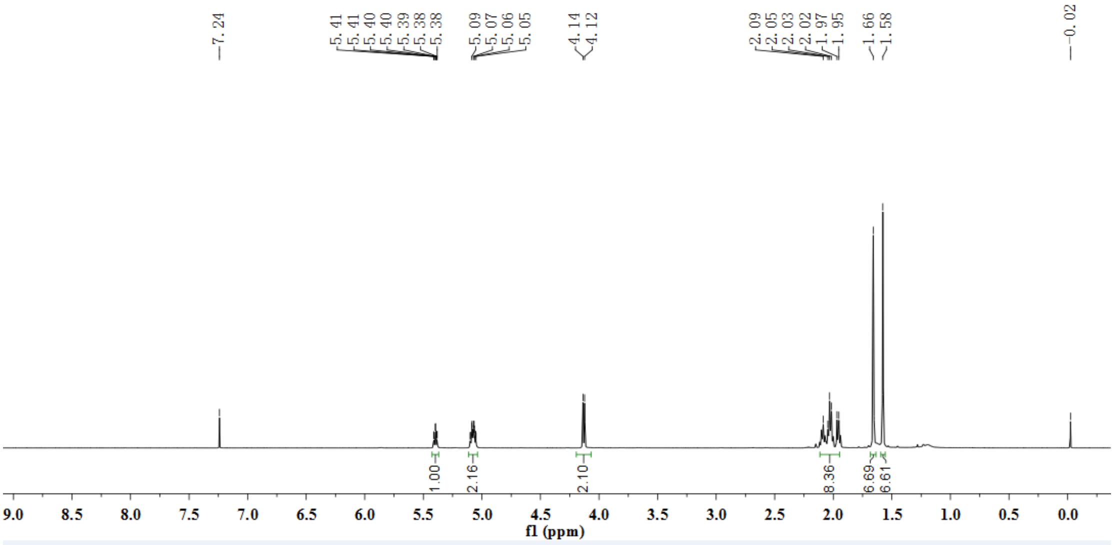

Fig S26. ^{1}H NMR of (*E*,*E*)-farnesol in $CDCl_3$ at 500 MHz.

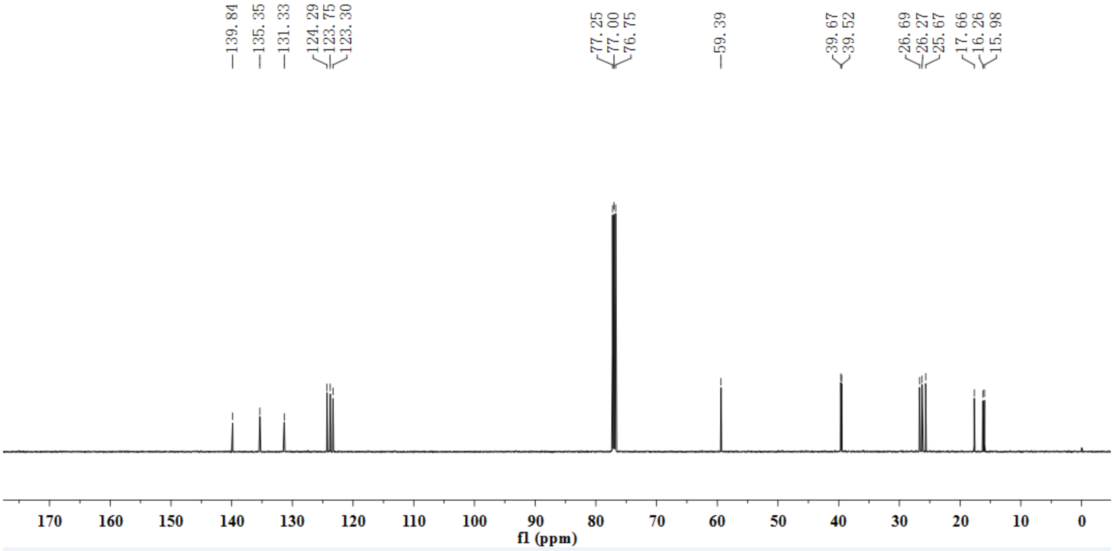

Fig S27. ^{13}C NMR of (*E*,*E*)-farnesol in $CDCl_3$ at 125 MHz.

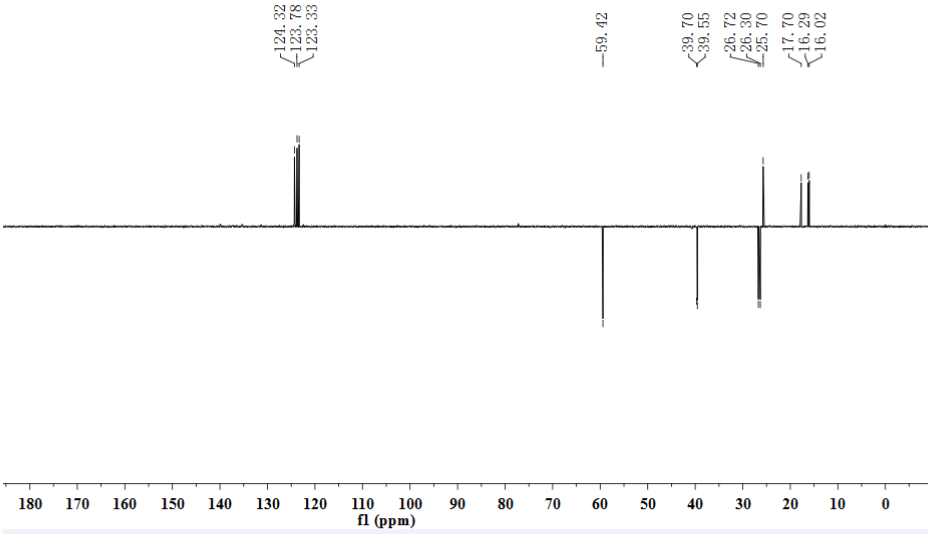

Fig S28. DEPT NMR of (*E*,*E*)-farnesol in $CDCl_3$ at 125 MHz.
